# Effectiveness of upper limb functional electrical stimulation after stroke for the improvement of activities of daily living and motor function: a systematic review and meta-analysis

**DOI:** 10.1186/s13643-017-0435-5

**Published:** 2017-02-28

**Authors:** John Eraifej, William Clark, Benjamin France, Sebastian Desando, David Moore

**Affiliations:** 1 School of Medicine, College of Medical and Dental Sciences, University of Birmingham, Edgbaston, Birmingham, B15 2TT UK; 2 Institute of Applied Health Research, College of Medical and Dental Sciences, University of Birmingham, Edgbaston, Birmingham, B15 2TT UK

**Keywords:** Functional electrical stimulation, Upper limb, Stroke, Systematic review, Meta-analysis, neurorehabilitation

## Abstract

**Background:**

Stroke can lead to significant impairment of upper limb function which affects performance of activities of daily living (ADL). Functional electrical stimulation (FES) involves electrical stimulation of motor neurons such that muscle groups contract and create or augment a moment about a joint. Whilst lower limb FES was established in post-stroke rehabilitation, there is a lack of clarity on the effectiveness of upper limb FES. This systematic review aims to evaluate the effectiveness of post-stroke upper limb FES on ADL and motor outcomes.

**Methods:**

Systematic review of randomised controlled trials from MEDLINE, PsychINFO, EMBASE, CENTRAL, ISRCTN, ICTRP and ClinicalTrials.gov. Citation checking of included studies and systematic reviews. Eligibility criteria: participants > 18 years with haemorrhagic/ischaemic stroke, intervention group received upper limb FES plus standard care, control group received standard care. Outcomes were ADL (primary), functional motor ability (secondary) and other motor outcomes (tertiary). Quality assessment using GRADE (Grading of Recommendations Assessment, Development and Evaluation) criteria.

**Results:**

Twenty studies were included. No significant benefit of FES was found for objective ADL measures reported in six studies (standardised mean difference (SMD) 0.64; 95% Confidence Interval (CI) [−0.02, 1.30]; total participants in FES group (*n*) = 67); combination of all ADL measures was not possible. Analysis of three studies where FES was initiated on average within 2 months post-stroke showed a significant benefit of FES on ADL (SMD 1.24; CI [0.46, 2.03]; *n* = 32). In three studies where FES was initiated more than 1 year after stroke, no significant ADL improvements were seen (SMD −0.10; CI [−0.59, 0.38], *n* = 35).

Quality assessment using GRADE found very low quality evidence in all analyses due to heterogeneity, low participant numbers and lack of blinding.

**Conclusions:**

FES is a promising therapy which could play a part in future stroke rehabilitation. This review found a statistically significant benefit from FES applied within 2 months of stroke on the primary outcome of ADL. However, due to the very low (GRADE) quality evidence of these analyses, firm conclusions cannot be drawn about the effectiveness of FES or its optimum therapeutic window. Hence, there is a need for high quality large-scale randomised controlled trials of upper limb FES after stroke.

**Trial Registration:**

PROSPERO: CRD42015025162, Date:11/08/2015

**Electronic supplementary material:**

The online version of this article (doi:10.1186/s13643-017-0435-5) contains supplementary material, which is available to authorized users.

## Background

Stroke is defined as a clinical syndrome characterised by rapidly developing focal or global disturbance in cerebral function lasting more than 24 h or leading to death due to a presumed vascular cause [1]. Globally, approximately 16 million people have a stroke each year [2] and in the UK, first-ever stroke affects about 230 people per 100,000 population each year [3]. Stroke represents a cost to the UK economy of approximately £9 billion annually, of which £1.33 billion results from productivity losses [4].

Stroke often leads to significant impairment of upper limb function and is associated with decreased quality of life in all domains except for mobility [5]. Few patients attain complete functional recovery [6]; this deficit impairs performance of activities of daily living (ADL), including self-care and social activities [7, 8]. ADL reflect the level of functional impairment in daily life and are therefore the most clinically relevant outcome measures in assessing recovery after stroke [9].

Functional electrical stimulation (FES) was well established as an intervention for motor rehabilitation. FES is the electrical stimulation of motor neurons such that muscle groups are stimulated to contract and create/augment a moment about a joint [2]. Transcutaneous electrodes offer the most immediate and clinically viable treatment option as they are non-invasive and may permit home-based treatment.

There are various terms used in the literature to describe different forms of electrical stimulation, often inconsistently. Some authors define FES as electrical stimulation applied to a subject which causes muscle contraction. This passive modality is also referred to as neuromuscular electrical stimulation [10]. Others define FES as electrical stimulation applied during a voluntary movement [4]. This definition acknowledges the volitional component of physical rehabilitation and was used in this systematic review. The distinction is important because neuroimaging studies have identified different cortical mechanisms according to stimulation type [11–13]. Indeed, perfusion to the ipsilesional sensory-motor cortex and cortical excitability were increased with FES when compared to passive modalities of electrical stimulation [12–14]. These findings could indicate greater potential for volitional FES to induce neuroplasticity. This is believed to play an important role in neurorehabilitation [15] and is a key objective of post-stroke functional recovery [16].

FES has been widely researched for post-stroke lower limb rehabilitation; several systematic reviews [17–19] and national guidelines [20, 21] exist. Improvement in upper limb function is central to post-stroke rehabilitation as it positively affects ADL and quality of life [22]. Yet, there is still a lack of clarity on the effectiveness of FES in post-stroke upper limb rehabilitation [23] despite systematic reviews having been undertaken [24–28]. In part, this is due to methodological limitations [27, 28] or the outdated nature of some existing reviews [24–26]. The latter was highlighted by a recent Cochrane overview of reviews calling for an up-to-date review and meta-analysis of randomised controlled trials (RCTs) related to electrical stimulation [29]. A more recent systematic review found a significant improvement in motor outcomes with upper limb FES [27]. However, this was based on a single meta-analysis that combined ADLs with upper limb-specific measures of functional motor ability, including studies where results were at risk of performance bias (intervention groups receiving greater duration of treatment than control groups) [27]. Another found no improvement in motor function when FES was applied within 6 months of stroke [28]. However, this predominantly included studies that applied electrical stimulation in the absence of volitional muscle contraction, confounding interpretation of the results. This inconsistency is reflected in the 2016 guidelines set by the Royal College of Physicians which recommends FES only in the context of clinical trials as an adjunct to conventional therapy [21].

This systematic review aims to elucidate the effectiveness of upper limb FES compared to standard therapy in improving ADL, in addition to motor outcomes, post-stroke. It represents an important addition to the literature that focuses on the use of volitional FES and, for the first time, distinguishes its effect on clinically relevant patient outcomes from surrogate markers of patient rehabilitation. This includes analyses based on patient sub-groups defined by the time after stroke at which FES was initiated.

## Methods

This systematic review was registered a priori on PROSPERO (CRD42015025162) [30] and was reported in accordance with the Preferred Reporting Items for Systematic Reviews and Meta-analysis (PRISMA) statement (see Additional file 1) [31].

### Search strategy

A systematic search of MEDLINE (Ovid), PsychINFO (Ovid), EMBASE (Ovid) and Cochrane Central Register of Controlled Trials databases from inception to 06/09/2015 was undertaken using a combination of free text and index terms for stroke, FES and upper limb. An example strategy is in Appendix 1. The following ongoing trial databases were also searched: International Standard Randomised Controlled Trials Number Registry, WHO International Clinical Trials Registry Platform and ClinicalTrials.gov. Citation checking was carried out on studies included in this review and existing systematic reviews to identify any further studies. Authors were contacted twice by email for original data where published study data was insufficient as to allow data analysis. Non-English language articles were translated where possible.

### Study selection

Inclusion criteria: population: patients >18 years diagnosed with ischaemic or haemorrhagic stroke. Intervention: intervention group receive transcutaneous FES applied to the peripheral nervous system of the upper limb defined as (a) applied to the skin externally and (b) during voluntary movement in addition to standard post-stroke rehabilitative therapy. Comparator: control groups receive standard post-stroke rehabilitative therapy alone, no between group differences other than the stimulation. Outcomes: ADL/motor outcomes recorded. Study design: RCTs and cross-over studies (only if randomised and controlled, such that first phase is equivalent to an RCT).

Exclusion criteria: (1) previous FES therapy in intervention or control group. (2) Other type of electrical stimulation used in intervention or control group. No other restrictions were placed on patient age, sex, ethnicity, time since stroke, baseline functional ability, publication date or language.

Two reviewers independently screened each title and abstract for relevance. Full texts of relevant articles were retrieved and assessed independently by two reviewers against the selection criteria. Disagreements between reviewers were discussed, and a third reviewer consulted if required.

### Outcome classification

Primary outcomes were those measures which directly assessed ADL. Secondary outcomes were those measures which assessed performance of a task that is not classified as an activity of daily living such as grasping and moving a cube. These were regarded as good surrogate outcomes of ADL and as such they were termed ‘functional motor ability’. Tertiary outcomes were any other measure of motor outcome: muscle tone, force generation, distance reached and range of active movement. Tertiary outcomes are regarded as poor surrogate outcomes which may not correlate with ADL. See Table S1 (Additional file 2) for full definitions of individual measurement instruments.

### Data extraction, risk of bias and quality assessment

Participant baseline characteristics, FES parameters and relevant outcome data at all reported time points were extracted; for cross-over studies, data from the first phase only were extracted. Data were extracted and the Cochrane Collaboration’s Tool for Assessing Risk of Bias was applied to all included studies by two reviewers independently; overall risk of bias judgement made based on most frequently cited risk across the seven categories. Quality assessment was performed using GRADE (Grading of Recommendations Assessment, Development and Evaluation) criteria. Risk of bias and quality assessment information was considered in interpretation of findings.

### Analysis

For each outcome, data were collated and assessed for suitability for meta-analysis. Care was taken to avoid double-counting of control group participants in meta-analyses. Meta-analysis was undertaken using a random effects model due to an underlying assumption that, although studies were similar, they would be representative of a distribution of effects on the outcome rather than represent a single underlying effect. Where the same outcome was measured using different but comparable tools on a continuous scale, standardised mean difference was used. Mean difference was used elsewhere. The *I* ^2^ was reported as a measure of heterogeneity, as well as the 95% confidence interval. Comparable tools were regarded to be those classified as primary outcomes and separately those classified as secondary outcomes. Tertiary outcome classification includes several types of measures which are not all comparable.

Study results reported as median, and interquartile ranges (IQR) were not included in meta-analyses. The shortest follow-up time post-stroke was used for analysis, for the purposes of consistency, as this was the most frequently reported time point. Where data were only represented graphically in papers, estimates were taken. Data that could not be incorporated into meta-analyses are reported narratively. Meta-analyses and forest plots were produced with Revman (version 5.35, Cochrane Collaboration). Separate analyses were undertaken for mean time post-stroke at which FES was initiated (less than 2 months; greater than 1 year).

## Results

### Included studies

**Fig. 1:**
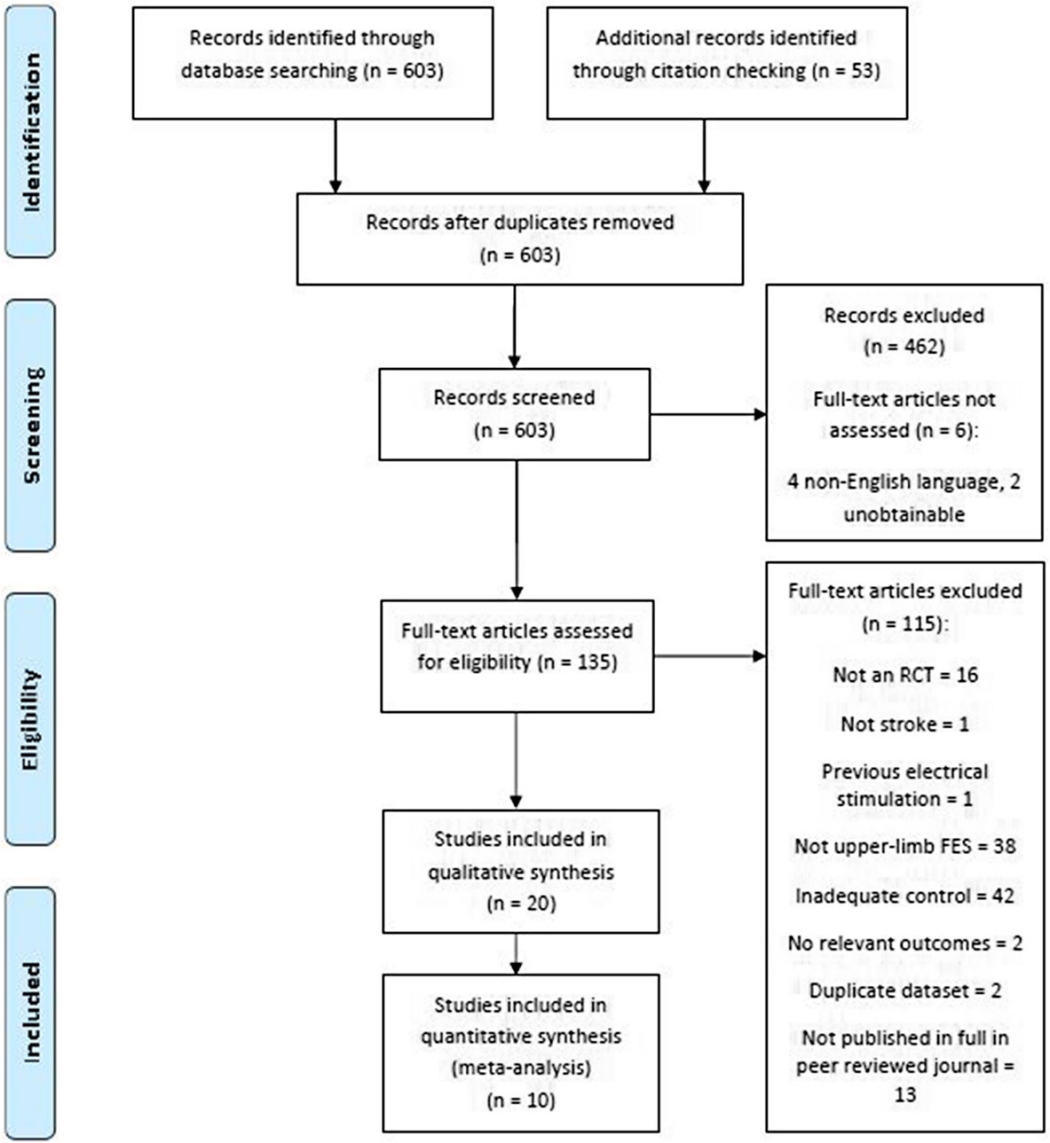
Flow diagram for included studies

The search strategy identified 603 records for screening; 135 studies proceeded to selection, of which two non-English language studies were translated. Twenty RCTs met the inclusion criteria. Six studies could not be assessed (Fig. 1). Details of excluded studies are in Appendix 2. Five possibly relevant ongoing clinical trials were identified (Appendix 3).

Details of the 20 included RCTs^A1-A20^ are presented in Table S2 (Additional file 2).

### Population

The included studies contained a total of 431 participants in the relevant intervention and control groups. Maximum group size was 28 participants and nine studies^A2-A5,A8,A12,A14,A15,A18^ included fewer than ten participants in either group. For studies which reported these measures, mean participant age was 60 (range: 45.5–66.4) and mean gender distribution was 68.8% male (range: 44.4–88.2). The mean time since stroke (across 15 studies) was 2.87 years with considerable variation: five studies^A12,A13,A16,A19,A20^ reporting mean times less than 2 months, five^A4,A9,A15,A17,A18^ reporting mean times between 1 and 3 years and six^A1,A2,A5-A7,A14^ reporting mean times over 3 years. Note one study^A16^ reported that all patients were treated within 60 days of stroke onset, hence mean time since stroke was less than 2 months, but it did not report a specific mean time. Of the 13 studies^A2,A4-A8,A10,A12-A16,A20^ which reported stroke site, 49.0% of participants (range: 9.1–75.0) had a left hemisphere stroke (all means are weighted by participant number in studies). There was also variation within and between studies in the severity of post-stroke impairment.

### Interventions and comparators

Two hundred thirty-eight participants received FES. For studies which reported FES parameters frequency ranged 20–50 Hz, peak current ≤ 70 mA and duration of stimulation from 3 to 10 s. Muscles stimulated included deltoid, triceps and the wrist and finger extensors/flexors.

One hundred ninety-three participants received a control treatment. Both intervention and control groups received standard care, which varied between studies but typically included physiotherapy, occupational therapy, task-based activities or other exercise-based interventions. In addition, three studies utilised orthoses^A1,A6,A16^, one utilised botulinum toxin^A7^ and one utilised mirror therapy^A20^. Three control groups also received sham FES, where a stimulation device delivered either no current at all or a sub-threshold current^A6,A7,A18^. Within all included studies control and intervention groups received equivalent total therapy durations, median session duration is 45 min, minimising the risk of performance bias.

### Risk of bias and quality assessment

Table S3 (Additional file 2) details the full critical appraisal information.

Included studies span a range of methodological quality. Nine studies were considered low risk of bias ^A5,A7,A9,A13,A14,A16-A19^. One study was considered high risk^A8^. The remaining ten studies^A1-A5,A10-A12,A15,A20^ were considered at an overall unclear risk of bias. Only the sham controlled studies^A6,A7,A18^ were considered to have adequate participant blinding.

Quality assessment using GRADE criteria found very low quality evidence in all analyses performed as a result of the heterogeneity, low participant numbers and lack of blinding in most studies.

### Activities of daily living

**Fig. 2:**
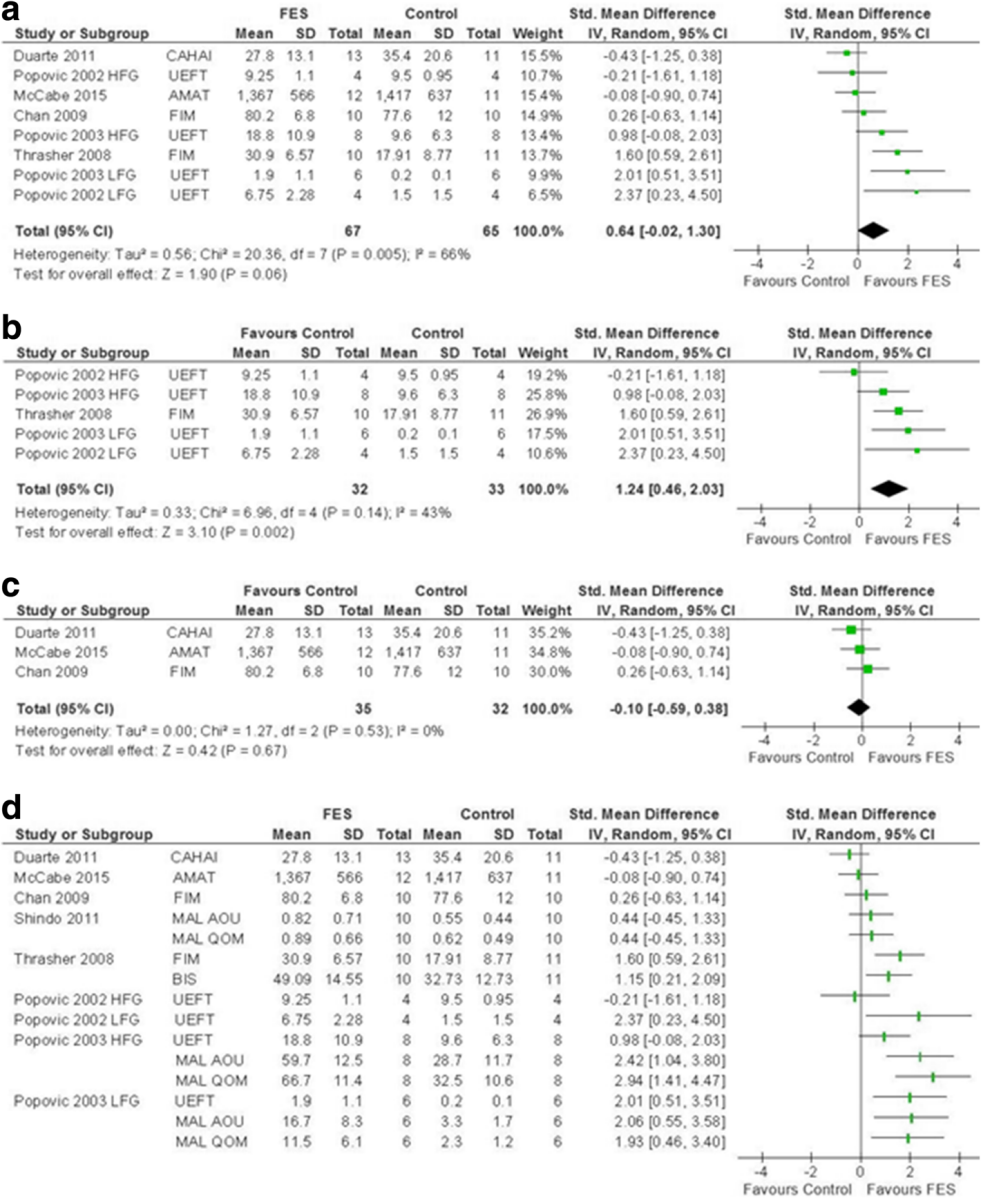
SMD (95% CI) of functional electrical stimulation (FES) vs control on activities of daily living. **a** Non-patient recall based measures of ADL. **b** FES initiated within 2 months of stroke. **c** FES initiated after 1 year of stroke. **d** Visual representation of all ADL measures. *AMAT* Arm Motor Ability Test, *CAHAI* Chedoke Arm and Hand Activity Inventory, *FIM* Functional Independence Measure, *UEFT* Upper Extremity Function Test, *HFG* higher functioning group, *LFG* lower functioning group

At least one measure of ADL (e.g. dressing and grooming) was reported by nine studies^A6-A8,A10-A13,A16,A19^. Seven studies provided data suitable for meta-analysis; of the other two, one provided insufficient data^A8^ and the other medians and interquartile ranges^A10^. Meta-analysis of results obtained through objectively assessed measures of ADL^A6,A7,A11-A13,A19^ was carried out separately from those that relied on patient recall, which may be at risk of recall bias. No difference was found between FES and control groups for the objectively obtained measures (SMD 0.64; CI [−0.02, 1.30]; *I* ^2^ = 66%) (Fig. 2a). Sensitivity analysis demonstrated that this effect was conserved when only sham-controlled studies were included in meta-analysis (Additional file 3: Figure S1).

An analysis of studies where FES was initiated in the acute phase^A12,A13,A19^ (mean time post-stroke less than 2 months) showed a significant benefit of FES (SMD 1.24; CI [0.46, 2.03]; *I* ^2^ = 43%) and moderate statistical heterogeneity (Fig. 2b). In contrast, where FES was initiated more than 1 year (mean time) after stroke^A6,A7,A11^, no significant improvements were seen (SMD −0.10; CI [−0.59, 0.38]; *I* ^2^ = 0%) (Fig. 2c).

Francisco et al. (1998)^A8^, which could not be included in this meta-analysis for reasons outlined above, also initiated FES within 2 months and reported a statistically significant improvement in functional independence measure (FIM). Similarly, Mangold et al. (2009) ^A10^ reported a significant improvement in extended Barthel index hand function subscore; patients were also treated on average within 2 months post-ictus.

Visual representation of all of the ADL data for meta-analysis is shown in Fig. 2d. No summary estimate is given due to inclusion of multiple ADL scales within several of the included studies. To combine these data in a meta-analysis, an arbitrary choice would have been made on which ADL scales to use from studies that use more than one measure of ADL. The figure indicates variable effect of FES although there seems to be a preponderance of data favouring a positive or no overall benefit.

### Functional improvement

**Fig. 3:**
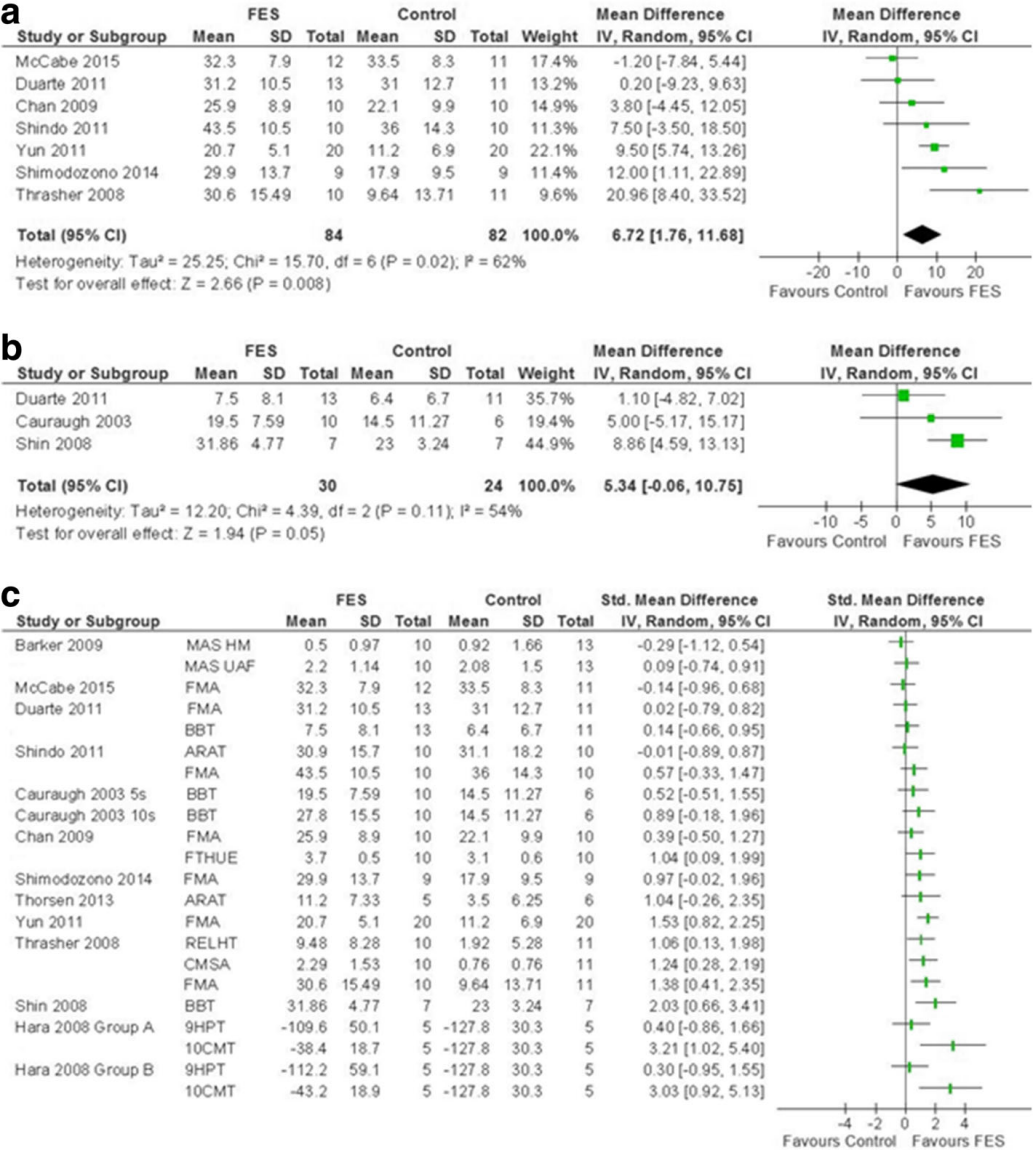
SMD (95% CI) of functional electrical stimulation (FES) vs control on secondary outcomes (functional motor recovery). **a** FMA. **b** BBT. **c** Visual representation of all secondary outcome measures. *MAS HM* Motor Assessment Scale Hand Movements, *MAS UAF* Motor Assessment Scale Upper Arm Function, *FMA* Fugl-Meyer Assessment, *BBT* Box and Block Test, *ARAT* Action Research Arm Test, *FTHUE* Functional Test for the Hemiparetic Upper Extremity, *RELHT* Rehabilitation Engineering Laboratory Hand Test (Block subscore shown here), *CMSA* Chedoke McMasters Stroke Assessment, *9HPT* Nine Hole Peg Test, *10CMT* Ten Cup Moving Test, *5s* 5-second stimulation pulse duration, *10s* 10-second stimulation pulse duration

Measures of functional improvement which do not incorporate ADL, were reported by 17 studies^A1-11,A14-A20^ (of which 4 presented data graphically^A2,A3,A5,A19^, 2 only reported narrative results^A2,A17^ and 2 reported medians and IQR^A5,A10^). Many tools were used in these studies to measure functional improvements. The findings are displayed in a forest plot for visual comparison (Fig. 3c) identifying no consistent trend across secondary outcomes. Some studies showed individual improvement^A6,A9,A15,A19,A20^. Again, similarly to ADL analyses, secondary outcome data were not pooled because where studies utilised more than one scale in this category, an arbitrary choice would have to be made on which to include to avoid double counting of participants.

Separate analysis with pooled totals was performed for two of the tools: Fugl-Meyer Assessment (FMA) and Box and Block Test (BBT). Meta-analysis of seven studies reporting FMA^A6,A7,A11,A14,A16,A19,A20^ (Fig. 3a) showed a statistically significant improvement in upper limb motor function with FES corresponding to a clinically important difference (MD 6.72; CI [1.76, 11.68], *I* ^2^ = 62%). One study, Francisco 1998, could not be included in this meta-analysis and individually reported significant FMA improvement with FES^A8^. Further analysis based on mean time since stroke demonstrated a significant improvement in FMA where FES was initiated within 2 months after stroke (MD 11.11; CI [5.07, 17.16]; *I* ^2^ = 37%)^A16,A19,A20^. In studies where FES was initiated over 1 year (mean time) there was no significant improvement (MD 2.75; CI [−2.46, 7.95]; *I* ^2^ = 32%) (Additional file 4: Figure S2) ^A6,A7,A11,A14^. Sensitivity analysis undertaken demonstrated that meta-analysis of sham-controlled studies did not favour FES; it should be noted that these studies initiated therapy after 1 year from stroke (mean) (Additional file 5: Figure S3).

Meta-analysis of BBT results showed no significant improvement with FES^A5,A7,A15^ (MD 5.34; CI [−0.06, 10.75]; *I* ^2^ = 54%) (Fig. 3b). FES was initiated on average more than 1 year after the stroke in these studies. The results of the studies that could not be included in this meta-analysis^A2-A4^ showed mixed results.

### Other motor outcomes

**Fig. 4:**
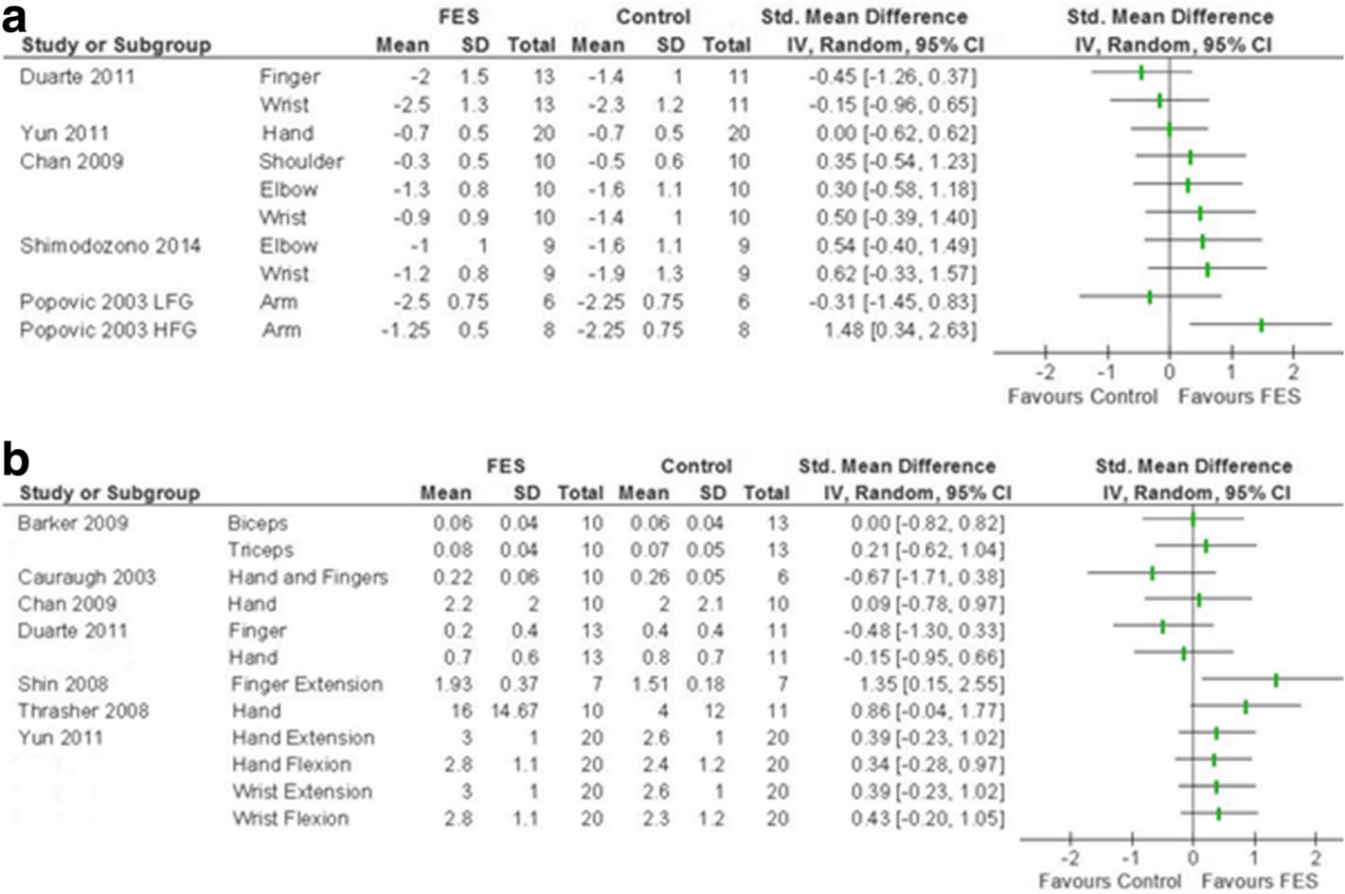
Visual representation of SMD (95% CI) of functional electrical stimulation (FES) vs control on tertiary outcomes. **a** Modified Ashworth Scale, upper limb component presented. **b** Force generation, muscle group/movement presented

These outcomes are assessed using tests that do not directly measure participant function but may contribute towards participant function in daily life, e.g. muscle tone or strength. These were reported in 14 studies^A1-A7,A9,A10,A13-A15,A19,A20^, of which 2 reported median and IQR^A5,A10^, and a further two presented no SD^A2,A3^. Five of the seven studies that measured Modified Ashworth Scale (a measure of muscle tone) provided sufficient information for analysis^A6,A7,A10,A13,A14^ (Fig. 4a). Many of these reported muscle-specific tone, hence a quantitative meta-analysis could not be performed. Seven of the nine studies that reported force generation provided sufficient information for analysis^A1,A4,A6,A7,A15,A19,A20^. However, due to frequent reporting in muscle or movement-specific subscales, this could only be displayed as a visual representation (Fig. 4b). There was no clear trend observed. There was insufficient data to analyse distance reached or range of active movement.

## Discussion

The aim of this systematic review was to evaluate the effect of post-stroke upper limb FES on activities of daily living (ADL) and motor outcomes. The results bring new understanding of the effectiveness of FES on upper limb rehabilitation after stroke.

When FES was initiated within 2 months (mean time) of stroke, there was a significant improvement in ADL compared to the control group. No benefit was found when FES was initiated over 1 year since stroke. Although there was substantial heterogeneity in the analysis of the 1 year post-stroke subgroup, these findings fit with studies which have found that the majority of motor recovery after stroke occurs in the initial few months [32–34]. However, one must also interpret these findings in light of the risk of bias of included studies. All studies in less than two month group lack adequate participant blinding, whilst two out of the three included studies in the one year group used sham treatment. Furthermore, the overall evidence quality, assessed using GRADE criteria, was very low as a result of the substantial heterogeneity, low participant numbers and lack of blinding in most studies.

Meta-analysis performed on objective ADL measures, which are considered more reliable than self-reported measures, found no significant benefit of FES. Patient recall-based ADL measures may not reliably correlate with objective measures of patient activity and clinical improvement [35–38]. Human perception is influenced by many factors and cognitive biases [38], recall bias is one such factor that has been shown to result in large errors in patient reported outcomes [36].

Meta-analysis of FMA, the most commonly reported measurement instrument, showed a statistically significant benefit of FES corresponding to a moderate effect size. Additional analysis of FMA found a significant benefit for FES applied within 2 months of stroke but not for FES applied 1 year or more after stroke. Most included studies in these analyses were not adequately blinded and overall evidence quality was very low.

Meta-analysis of BBT results found no benefit from FES, but this could have been masked by the low group numbers: only 30 patients in total for the intervention groups and 24 in control groups. Indeed all studies included in this review had small group sizes, thus it is likely that these studies would lack the power necessary to demonstrate a small increase in upper limb motor function. Even a small improvement in function may be clinically significant, since upper limb function is so important for daily activities [39]. An alternative explanation is that all studies which reported BBT applied FES 1 year or more after stroke, so the lack of improvement in BBT with FES could also reflect the fact that FES was not applied during the optimum therapeutic window. However, caution is needed when drawing conclusions about the optimum time window for FES post-stroke given the very low quality of evidence in the above analyses.

It is possible that FES is beneficial only when applied using certain stimulation parameters or when applied to a specific patient population. Indeed, variation in FES parameters including current, frequency, duration of stimulation and also in baseline function of participants both between and within studies were noted. It appears that there are no agreed stimulation parameters, and it is likely that none of the included studies employed exactly the same stimulation protocol. Potential benefits could thus be hidden among the inter-study variability between studies in this systematic review. This variability in FES parameters could influence results in this review and may be a contributing factor to the heterogeneity in the analyses.

To our knowledge, four systematic reviews have previously attempted to evaluate the effectiveness of FES for upper limb rehabilitation after stroke [24–27].

Van Peppen et al. [25] found no improvement with FES in tertiary outcomes, specifically strength or dexterity. Bolton et al. [26] carried out a meta-analysis on five electromyography (EMG)-triggered neuromuscular stimulation studies and found an improvement across secondary outcomes as defined by the present systematic review. Meta-analysis by Meilink et al. [24] found no significant improvement in BBT. However, whilst the current review found a significant improvement in FMA, Meilink et al. did not. This discrepancy could be explained by the small meta-analysis group size of three studies in Meilink et al. compared to the eight studies here. Howlett et al. [24] conducted the most recent relevant systematic review. Whilst they found a significant improvement with upper limb FES compared to control, this finding was based on a single meta-analysis which combined primary and secondary outcomes as defined above. These outcomes are arguably not comparable. Moreover, such an approach prevents primary, secondary and tertiary outcomes from being independently assessed. To date, no systematic review has assessed the therapeutic window of upper limb FES application post-stroke.

### Strengths and limitations

This systematic review is the most comprehensive and provides a much needed evaluation of upper limb-specific FES after stroke, which was missing from the literature. Analyses were performed, in accordance with the a priori protocol, enabling important conclusions about the use of FES in clinical practice to be drawn.

99% of the articles identified by our search strategy were assessed. However, it was not possible to translate four studies that were not in the English language and two articles could not be found after extensive attempts to locate them (see Appendix 2).

Meta-analyses gave rise to certain limitations. Firstly, included studies utilised many different measurement instruments and only a minority were employed by more than a few studies. Secondly, many studies used multiple measurement instruments for each outcome, e.g. several scales which measure ADL in a single study. As such, it was not appropriate to combine data in single meta-analyses. Thirdly, one study contained multiple relevant intervention groups but only a single control group for comparison^A4^; the intervention group most similar to the other included studies was selected.

The small number and distribution of studies limited potential for formal between group comparisons in form of subgroup analyses. In addition, analyses on severity of stroke and stimulation parameters, which we had intended in our protocol, were not possible due to methodological variability.

All included studies, with the exception of one^A8^ which could not be included in quantitative analysis, were considered at low or unclear risk of bias. Inadequate participant blinding was an issue in most studies.

### Recommendations for clinical practice and research

This systematic review found insufficient evidence of clinical benefit to support routine use of FES in clinical practice; however, this may reflect a lack of high quality trials in the field which strongly supports the need for FES use in clinical trials.

This systematic review highlights the need for large RCTs in order to ensure homogeneity among groups and to have sufficient power to identify small functional improvements. Randomised triple-blinded controlled trials, where comparator groups receive sham treatment (subthreshold stimulation), are recommended as the optimal study design. An RCT that includes two intervention groups with FES applied at two different time points after stroke (e.g. 2 months and 1 year) could help clarify the optimal therapeutic window for FES. Future research should also attempt to identify the optimal FES parameters in order to standardise FES treatment for future studies.

It is advisable that prospective RCTs in this field use an agreed core outcome set unless there is a clear justification to use alternative measures [40] because the use of different but comparable measurement instruments limits the suitability of data for meta-analysis. Millar et al. are currently working on a core outcome set for upper limb rehabilitation after stroke to aid evidence-based clinical practice [41].

## Conclusions

FES is a promising therapy which could play a part in future stroke rehabilitation strategies. This review found a statistically significant benefit from FES applied within 2 months of stroke on our primary outcome of ADL. However, due to the very low (GRADE) quality evidence of these analyses, firm conclusions about the effectiveness of FES or its optimum therapeutic window cannot be drawn. Hence, there is a need for high quality large-scale randomised controlled trials of upper limb FES after stroke.

## Appendix 1

### Search Strategy

The following is the search strategy used to search databases on Ovid. The search terminology was adapted to meet Cochrane search requirements but remained otherwise unchanged. The same search strategy was used to identify both primary studies and systematic reviews, but the databases searched differed as outlined in the Methods section.

1. electrostimulat*
2. electric* stimulat*
3. electrotherap*
4. transcutaneous adj5 stimulat*
5. neurostimulat*
6. Electric stimulation/
7. 1 OR 2 OR 3 OR 4 OR 5 OR 6
8. Stroke/
9. stroke*
10. CVA
11. cerebrovasc*
12. brain* or cerebr*
13. isch?em* or thromb* or embol* or infarct*
14. 12 AND 13
15. h?emorrhage or h?ematoma or bleed
16. 12 AND 15
17. 8 OR 9 OR 10 OR 11 OR 14 OR 16
18. upper limb*
19. shoulder*
20. arm*
21. forearm*
22. wrist*
23. hand*
24. Upper Extremity/
25. finger*
26. digit*
27. 18 OR 19 OR 20 OR 21 OR 22 OR 23 OR 24 OR 25 OR 26
28. 7 AND 17 AND 27
29. Randomized Controlled Trials as Topic/
30. random allocation/
31. Controlled Clinical Trials as Topic/
32. control groups/
33. clinical trials as topic/
34. double-blind method/
35. single-blind method/
36. Placebos/
37. placebo effect/
38. Research Design/
39. Program Evaluation/
40. randomized controlled trial.pt.
41. controlled clinical trial.pt.
42. clinical trial.pt.
43. (random* or RCT or RCTs).tw.
44. (controlled adj5 (trial* or stud*)).tw.
45. (clinical* adj5 trial*).tw.
46. ((control or treatment or experiment* or intervention) adj5 (group* or subject* or patient*)).tw.
47. (quasi-random* or quasi random* or pseudo-random* or pseudo random*).tw.
48. ((control or experiment* or conservative) adj5 (treatment or therapy or procedure or manage*)).tw.
49. ((singl* or doubl* or tripl* or trebl*) adj5 (blind* or mask*)).tw.
50. placebo*.tw.
51. sham.tw.
52. (assign* or allocat*).tw.
53. controls.tw.
54. or/29-53
55. 28 and 54
56. exp animals/not humans.sh.
57. 55 not 56

## Appendix 2

### Excluded studies list

**Table 1:** ᅟ

Study	Not an RCT	Not stroke	Previous stimulation	Not upper limb FES	Inadequate control	No relevant outcomes	Duplicate dataset	Not published in full in peer reviewed journal
(1) Ottawa panel evidence-based clinical practice guidelines for post-stroke rehabilitation. Topics in Stroke Rehabilitation Spr 2006; 13(2):1–269.	x							
(2) Alon G, Levitt AF, McCarthy PA. Functional electrical stimulation (FES) may modify the poor prognosis of stroke survivors with severe motor loss of the upper extremity: a preliminary study. American Journal of Physical Medicine & Rehabilitation 2008 Aug; 87(8):627–636.					x			
(3) Alon G, Levitt AF, McCarthy PA. Functional electrical stimulation enhancement of upper extremity functional recovery during stroke rehabilitation: a pilot study. Neurorehabilitation and Neural Repair 2007 May–Jun; 21(3):207–215.					x			
(4) Alon G. Defining and measuring residual deficits of the upper extremity following stroke: a new perspective. Topics in Stroke Rehabilitation 2009 May–Jun; 16(3):167–176.					x			
(5) Alon G, Ring H. Gait and hand function enhancement following training with a multi-segment hybrid-orthosis stimulation system in stroke patients. J Stroke Cerebrovasc Dis 2003; 12(5):209–216.					x			
(6) Armagan O, Tascioglu F, Oner C. Electromyographic biofeedback in the treatment of the hemiplegic hand: a placebo-controlled study. Am J Phys Med Rehabil 2003 Nov; 82(11):856–861.				x				
(7) Au-Yeung SS, Hui-Chan CW. Electrical acupoint stimulation of the affected arm in acute stroke: a placebo-controlled randomized clinical trial. Clin Rehabil 2014 Feb; 28(2):149–158.				x				
(8) Barker RN, Brauer SG, Carson RG. Training of reaching in stroke survivors with severe and chronic upper limb paresis using a novel nonrobotic device: a randomized clinical trial. Stroke 2008 Jun; 39(6):1800–1807.							x	
(9) Basmajian JV, Gowland C, Brandstater ME, Swanson L, Trotter J. EMG feedback treatment of upper limb in hemiplegic stroke patients: a pilot study. Arch Phys Med Rehabil 1982 Dec; 63(12):613–616.				x				
(10) Bhatt E, Nagpal A, Greer KH, Grunewald TK, Steele JL, Wiemiller JW, et al. Effect of finger tracking combined with electrical stimulation on brain reorganization and hand function in subjects with stroke. Experimental Brain Research 2007 Oct; 182(4):435–447.					x			
(11) Boespflug EL, Storrs JM, Allendorfer JB, Lamy M, Eliassen JC, Page S. Mean diffusivity as a potential diffusion tensor biomarker of motor rehabilitation after electrical stimulation incorporating task specific exercise in stroke: a pilot study. Brain imaging behav 2014 Sep; 8(3):359–369.					x			
(12) Bowman BR, Baker LL, Waters RL. Positional feedback and electrical stimulation: an automated treatment for the hemiplegic wrist. Archives of Physical Medicine and Rehabilitation 1979 Nov; 60(11):497–502.					x			
(13) Boyaci A, Topuz O, Alkan H, Ozgen M, Sarsan A, Yildiz N, et al. Comparison of the effectiveness of active and passive neuromuscular electrical stimulation of hemiplegic upper extremities: a randomized, controlled trial. International Journal of Rehabilitation Research 2013 Dec; 36(4):315–322.					x			
(14) Cauraugh JH, Coombes SA, Lodha N, Naik SK, Summers JJ. Upper extremity improvements in chronic stroke: coupled bilateral load training. Restorative Neurology and Neuroscience 2009; 27(1):17–25.					x			
(15) Cauraugh JH, Kim SB, Duley A. Coupled bilateral movements and active neuromuscular stimulation: intralimb transfer evidence during bimanual aiming. Neurosci Lett 2005 Jul 1–8; 382(1–2):39–44.					x			
(16) Cauraugh JH, Kim SB, Summers JJ. Chronic stroke longitudinal motor improvements: cumulative learning evidence found in the upper extremity. Cerebrovascular Diseases 2008; 25(1–2):115–121.					x			
(17) Celnik P, Hummel F, Harris-Love M, Wolk R, Cohen LG. Somatosensory stimulation enhances the effects of training functional hand tasks in patients with chronic stroke. Archives of Physical Medicine and Rehabilitation 2007 Nov; 88(11):1369–1376.				x				
(18) Celnik P, Paik NJ, Vandermeeren Y, Dimyan M, Cohen LG. Effects of combined peripheral nerve stimulation and brain polarization on performance of a motor sequence task after chronic stroke. Stroke 2009 May; 40(5):1764–1771.				x				
(19) Chae J, Bethoux F, Bohine T, Dobos L, Davis T, Friedl A. Neuromuscular stimulation for upper extremity motor and functional recovery in acute hemiplegia. Stroke 1998 May; 29(5):975–979.				x				
(20) Chae J, Page S, Delahanty M. Surface electrical stimulation does not improve hand function in subacute stroke. Stroke 2012; 43(2 Meeting Abstracts).								x
(21) Chantraine A, Baribeault A, Uebelhart D, Gremion G. Shoulder pain and dysfunction in hemiplegia: effects of functional electrical stimulation. Archives of Physical Medicine and Rehabilitation 1999 Mar; 80(3):328–331.					x			
(22) Church C, Price C, Pandyan AD, Huntley S, Curless R, Rodgers H. Randomized controlled trial to evaluate the effect of surface neuromuscular electrical stimulation to the shoulder after acute stroke. Stroke 2006 Dec; 37(12):2995–3001.				x				
(23) Church C, Curless R, Huntley S, Price C, Pandyan D, Rodgers H. Does surface neuromuscular electrical stimulation (sNMES) to the upper limb following acute stroke improve outcome? 12th European Stroke Conference 2003.								x
(24) Conforto AB, Cohen LG, dos Santos RL, Scaff M, Marie SK. Effects of somatosensory stimulation on motor function in chronic cortico-subcortical strokes. J Neurol 2007 Mar; 254(3):333–339.				x				
(25) Conforto AB, Ferreiro KN, Tomasi C, dos Santos RL, Moreira VL, Marie SK, et al. Effects of somatosensory stimulation on motor function after subacute stroke. Neurorehabilitation and Neural Repair 2010 Mar–Apr; 24(3):263–272.				x				
(26) Conforto AB, Kaelin-Lang A, Cohen LG. Increase in hand muscle strength of stroke patients after somatosensory stimulation. Ann Neurol 2002 Jan; 51(1):122–125.				x				
(27) Conforto AB, Fontes RLS, Ferreiro KN, Correia CER, Scaff M. Home-based peripheral nerve stimulation enhances effects of motor training in patients in the chronic phase after stroke. Cerebrovascular Diseases 2010 May 2010; 29:251.				x				
(28) Daly JJ, Rogers J, McCabe J, Monkiewicz M, Burdsall R, Pundik S. Recovery of actual functional tasks in response to motor learning, robotics, and functional electrical stimulation. Stroke 2010 01 Apr 2010; 41(4):e355–e356.								x
(29) Dorsch S, Ada L, Canning CG. EMG-triggered electrical stimulation is a feasible intervention to apply to multiple arm muscles in people early after stroke, but does not improve strength and activity more than usual therapy: a randomized feasibility trial. Clin Rehabil 2014 May; 28(5):482–490.					x			
(30) Dos Santos-Fontes RL, Ferreiro de Andrade KN, Sterr A, Conforto AB. Home-based nerve stimulation to enhance effects of motor training in patients in the chronic phase after stroke: a proof-of-principle study. Neurorehabilitation and Neural Repair 2013 Jul–Aug; 27(6):483–490.				x				
(31) Faghri PD, Rodgers MM, Glaser RM, Bors JG, Ho C, Akuthota P. The effects of functional electrical stimulation on shoulder subluxation, arm function recovery, and shoulder pain in hemiplegic stroke patients. Archives of Physical Medicine and Rehabilitation 1994 Jan; 75(1):73–79.					x			
(32) Fields RW. Electromyographically triggered electric muscle stimulation for chronic hemiplegia. Archives of Physical Medicine and Rehabilitation 1987 Jul; 68(7):407–414.					x			
(33) Fil A, Armutlu K, Atay AO, Kerimoglu U, Elibol B. The effect of electrical stimulation in combination with Bobath techniques in the prevention of shoulder subluxation in acute stroke patients. Clin Rehabil 2011 Jan; 25(1):51–59.					x			
(34) Fleming MK, Newham DJ, Roberts-Lewis SF, Sorinola IO. Self-perceived utilization of the paretic arm in chronic stroke requires high upper limb functional ability. Archives of Physical Medicine and Rehabilitation 2014 May; 95(5):918–924.				x				
(35) Fleming MK, Sorinola IO, RobertsLewis SF, Wolfe CD, Wellwood I, Newham DJ. The effect of combined somatosensory stimulation and task-specific training on upper limb function in chronic stroke: A double-blind randomized controlled trial. Neurorehabil Neural Repair Feb 2015; 29(2):143–152.				x				
(36) Fujiwara T. Effect of hybrid assistive neuromuscular dynamic stimulation (HANDS) therapy for functional recovery after stroke. Clinical Neurophysiology 2010 October 2010; 121:S49.								x
(37) Gabr U, Levine P, Page SJ. Home-based electromyography-triggered stimulation in chronic stroke. Clin Rehabil 2005 Oct; 19(7):737–745.					x			
(38) Gharib NMM, Aboumousa AM, Elowishy AA, RezkAllah SS, Yousef FS. Efficacy of electrical stimulation as an adjunct to repetitive task practice therapy on skilled hand performance in hemiparetic stroke patients: a randomized controlled trial. Clin Rehabil Apr 2015; 29(4):355–364.				x				
(39) Hara Y, Ogawa S, Muraoka Y. Hybrid power-assisted functional electrical stimulation to improve hemiparetic upper-extremity function. American Journal of Physical Medicine & Rehabilitation 2006 Dec; 85(12):977–985.							x	
(40) Hayward KS, Barker RN, Brauer SG, Lloyd D, Horsley SA, Carson RG. SMART Arm with outcome-triggered electrical stimulation: a pilot randomized clinical trial. Topics in Stroke Rehabilitation 2013 Jul–Aug; 20(4):289–298.					x			
(41) Heckmann J, Mokrusch T, Krockel A, Warnke S, Von ST, Neundorfer B. EMG-triggered electrical muscle stimulation in the treatment of central hemiparesis after a stroke. Eur J Phys Med Rehabil 1997; 7(5):138–141.					x			
(42) Hesse S, Reiter F, Konrad M, Jahnke MT. Botulinum toxin type A and short-term electrical stimulation in the treatment of upper limb flexor spasticity after stroke: a randomized, double-blind, placebo-controlled trial. Clin Rehabil 1998 Oct; 12(5):381–388.				x				
(43) Hesse S, Schmidt H, Werner C. Machines to support motor rehabilitation after stroke: 10 years of experience in Berlin. Journal of Rehabilitation Research and Development 2006 Aug–Sep; 43(5):671–678.					x			
(44) Hsu SS, Hu MH, Wang YH, Yip PK, Chiu JW, Hsieh CL. Dose-response relation between neuromuscular electrical stimulation and upper-extremity function in patients with stroke. Stroke 2010 Apr; 41(4):821–824.					x			
(45) Ikuno K, Kawaguchi S, Kitabeppu S, Kitaura M, Tokuhisa K, Morimoto S, et al. Effects of peripheral sensory nerve stimulation plus task-oriented training on upper extremity function in patients with subacute stroke: a pilot randomized crossover trial. Clin Rehabil 2012 Nov; 26(11):999–1009.	x							
(46) Inobe J-, Kato T. Effectiveness of finger equipped electrode (FEE)-triggered electrical stimulation in chronic stroke patients with severe hemiplegia. International Journal of Stroke 2010 October 2010; 5:290.	x							
(47) Inobe J, Kato T. Effectiveness of finger-equipped electrode (FEE)-triggered electrical stimulation improving chronic stroke patients with severe hemiplegia. Brain Injury Jan 2013; 27(1):114–119.	x							
(48) Joa KL, Kim WH, Min JH. The synergistic effects of mirror therapy and functional electrical stimulation on hand function in severe stroke patients. Cerebrovasc Dis 2013; 35 Suppl 3:588–589.								x
(49) Johansson BB, Haker E, von Arbin M, Britton M, Langstrom G, Terent A, et al. Acupuncture and transcutaneous nerve stimulation in stroke rehabilitation: a randomized, controlled trial. Stroke 2001 Mar; 32(3):707–713.				x				
(50) Karakus D, ErsoZ M, Koyuncu G, Turk D, Sasmaz FM, AkyuZ M. Effects of functional electrical stimulation on wrist function and spasticity in stroke: a randomized controlled study. Turkiye Fiziksel Tip ve Rehabilitasyon Dergisi 2013; 59(2):97–102.					x			
(51) Kawahira K, Shimodozono M, Etoh S, Kamada K, Noma T, Tanaka N. Effects of intensive repetition of a new facilitation technique on motor functional recovery of the hemiplegic upper limb and hand. Brain Inj 2010; 24(10):1202–1213.				x				
(52) Kim TH, In TS, Cho HY. Task-related training combined with transcutaneous electrical nerve stimulation promotes upper limb functions in patients with chronic stroke. Tohoku J Exp Med 2013; 231(2):93–100.				x				
(53) Kim JH, Lee B. Mirror therapy combined with biofeedback functional electrical stimulation for motor recovery of upper extremities after stroke: a pilot randomized controlled trial. Occupational Therapy International Jun 2015; 22(2):51–60.					x			
(54) Kimberley TJ, Lewis SM, Auerbach EJ, Dorsey LL, Lojovich JM, Carey JR. Electrical stimulation driving functional improvements and cortical changes in subjects with stroke. Experimental Brain Research 2004 Feb; 154(4):450–460.				x				
(55) King TI. The effect of neuromuscular electrical stimulation in reducing tone. Am J Occup Ther 1996; 50(1):62–64.					x			
(56) Knutson JS, Hisel TZ, Harley MY, Chae J. A novel functional electrical stimulation treatment for recovery of hand function in hemiplegia: 12-week pilot study. Neurorehabil Neural Repair 2009 Jan; 23(1):17–25.	x							
(57) Kobayashi H, Onishi H, Ihashi K, Yagi R, Handa Y. Reduction in subluxation and improved muscle function of the hemiplegic shoulder joint after therapeutic electrical stimulation. Journal of Electromyography and Kinesiology 1999 Oct; 9(5):327–336.				x				
(58) Koyuncu E, Nakipoglu-Yuzer GF, Dogan A, Ozgirgin N. The effectiveness of functional electrical stimulation for the treatment of shoulder subluxation and shoulder pain in hemiplegic patients: a randomized controlled trial. Disability and Rehabilitation 2010; 32(7):560–566.					x			
(59) Kraft GH, Fitts SS, Hammond MC. Techniques to improve function of the arm and hand in chronic hemiplegia. Archives of Physical Medicine and Rehabilitation 1992 Mar; 73(3):220–227.					x			
(60) Lai CJ, Wang CP, Tsai PY, Chan RC, Lin SH, Lin FG, et al. Corticospinal integrity and motor impairment predict outcomes after excitatory repetitive transcranial magnetic stimulation: a preliminary study. Arch Phys Med Rehabil 2015 Jan; 96(1):69–75.				x				
(61) Leandri M, Parodi CI, Corrieri N, Rigardo S. Comparison of TENS treatments in hemiplegic shoulder pain. Scand J Rehabil Med 1990; 22(2):69–71.						x		
(62) Leung J, Harvey LA, Moseley AM, Tse C, Bryant J, Wyndham S, et al. Electrical stimulation and splinting were not clearly more effective than splinting alone for contracture management after acquired brain injury: a randomised trial. Journal of Physiotherapy 2012; 58(4):231–240.					x			
(63) Li L, Yuan J, Zhang C. Clinical observation on hemiplegic patients with functional electric stimulation and intensive training on their lower extremities. Chinese Journal of Physical Medicine and Rehabilitation 2000; 22(1):18–19.				x				
(64) Li N, Tian FW, Wang CW, Yu PM, Zhou X, Wen Q, et al. Double-center randomized controlled trial on post-stroke shoulder pain treated by electroacupuncture combined with Tuina. Zhongguo zhen jiu [Chinese acupuncture and moxibustion] 2012; 32(2):101–105.				x				
(65) Liepert J, Restemeyer C, Munchau A, Weiller C. Motor cortex excitability after thalamic infarction. Clinical Neurophysiology 2005 Jul; 116(7):1621–1627.				x				
(66) Lin KC, Huang PC, Chen YT, Wu CY, Huang WL. Combining afferent stimulation and mirror therapy for rehabilitating motor function, motor control, ambulation, and daily functions after stroke. Neurorehabilitation and Neural Repair 2014 Feb; 28(2):153–162.				x				
(67) Lin Z, Yan T. Long-term effectiveness of neuromuscular electrical stimulation for promoting motor recovery of the upper extremity after stroke. J Rehabil Med 2011 May; 43(6):506–510.					x			
(68) Lin K, Chen Y, Huang P, Wu C, Huang W, Yang H, et al. Effect of mirror therapy combined with somatosensory stimulation on motor recovery and daily function in stroke patients: a pilot study. J Formos Med Assoc 2014 Jul; 113(7):422–428.				x				
(69) Linn SL, Granat MH, Lees KR. Prevention of shoulder subluxation after stroke with electrical stimulation. Stroke 1999 May; 30(5):963–968.					x			
(70) Linn SL, Grant MH, Crossan JS, Lees KR. The use of electrical stimulation for the prevention of shoulder subluxation post stroke. Physiotherapy 1995; 81(12):742.								x
(71) Lisinski P, Huber J, Samborski W, Witkowska A. Neurophysiological assessment of the electrostimulation procedures used in stroke patients during rehabilitation. Int J Artif Organs 2008 Jan; 31(1):76–86.	x							
(72) Liu J, You WX, Sun D. [Effects of functional electric stimulation on shoulder subluxation and upper limb motor function recovery of patients with hemiplegia resulting from stroke]. Di Yi Junyi Daxue Xuebao 2005 Aug; 25(8):1054–1055.					x			?
(73) Looned R, Webb J, Xiao ZG, Menon C. Assisting drinking with an affordable BCI-controlled wearable robot and electrical stimulation: a preliminary investigation. Journal of Neuroengineering and Rehabilitation 2014; 11:51.		x						
(74) Lum PS, Burgar CG, Van der Loos M, Shor PC, Majmundar M, Yap R. MIME robotic device for upper-limb neurorehabilitation in subacute stroke subjects: a follow-up study. J Rehabil Res Dev 2006 Aug–Sep; 43(5):631–642.				x				
(75) Makowski NS, Knutson JS, Chae J, Crago P. Variations in neuromuscular electrical stimulation's ability to increase reach and hand opening during voluntary effort after stroke. Conference Proceedings: …Annual International Conference of the IEEE Engineering in Medicine and Biology Society 2012; 2012:318–321.								x
(76) Malhotra S, Rosewilliam S, Hermens H, Roffe C, Jones P, Pandyan AD. A randomized controlled trial of surface neuromuscular electrical stimulation applied early after acute stroke: effects on wrist pain, spasticity and contractures. Clin Rehabil 2013 Jul; 27(7):579–590.				x				
(77) Mann G, Taylor P, Lane R. Accelerometer-triggered electrical stimulation for reach and grasp in chronic stroke patients: a pilot study. Neurorehabilitation and Neural Repair 2011 Oct; 25(8):774–780.	x							
(78) Mann GE, Burridge JH, Malone LJ, Strike PW. A pilot study to investigate the effects of electrical stimulation on recovery of hand function and sensation in subacute stroke patients. Neuromodulation 2005;8(3):193–202.					x			
(79) Mann GE, Malone LJ, Taylor PN, Burridge JH. A randomised controlled pilot study to investigate the effect of neuromuscular electrical stimulation on upper limb function and hand sensation following stroke. 1st Annual Conference of Fesnet 2002 2002.								x
(80) Mano TS, Terradez JRS, Tomas JM, Moral JCM, Fuente FT, Jose CC. Electrical stimulation in the treatment of the spastic hemiplegic hand after stroke: a randomized study. Spanish. Med Clin 2011; 137(7):297–301.					x			
(81) McDonnell MN, Hillier SL, Miles TS, Thompson PD, Ridding MC. Influence of combined afferent stimulation and task-specific training following stroke: a pilot randomized controlled trial. Neurorehabilitation and Neural Repair 2007 Sep-Oct; 21(5):435–443.				x				
(82) Meadmore K, Exell T, Freeman C, Kutlu M, Rogers E, Hughes AM, et al. Electrical stimulation and iterative learning control for functional recovery in the upper limb post-stroke. IEEE International Conference on Rehabilitation Robotics 2013 Jun; 2013:6650359.	x							
(83) Meadmore KL, Exell TA, Hallewell E, Hughes AM, Freeman CT, Kutlu M, et al. The application of precisely controlled functional electrical stimulation to the shoulder, elbow and wrist for upper limb stroke rehabilitation: a feasibility study. Journal of Neuroengineering and Rehabilitation 2014; 11:105.	x							
(84) Merletti R, Acimovic R, Grobelnik S, Cvilak G. Electrophysiological orthosis for the upper extremity in hemiplegia: feasibility study. Arch Phys Med Rehabil 1975 1975; 56(12):507–513.	x							
(85) Mohamed FCK, Prakash PNO, Ajith S. Efficacy of functional neuromuscular electrical stimulation (FNMES) in the improvement of hand functions in acute stroke survivals. Nitte University Journal of Health Science 2012; 2(4):16–21.					x			
(86) Mokrusch T. Treatment of stroke-induced spastic hemoparesis with EMG-triggered electrostimulation. German. Neurologie and Rehabilitation 1997; 3(2):82–86.								x
(87) Moniruzzaman M, Salek KM, Shakoor MA, Mia BA, Moyeenuzzaman M. Effects of therapeutic modalities on patients with post stroke shoulder pain. Mymensingh Medical Journal: MMJ 2010 Jan; 19(1):48–53.						x		
(88) Nakipoglu-Yuzer GF, Koyuncu E, Ozgirgin N. Effectiveness of functional electrical stimulation on upper extremity rehabilitation outcomes in patients with hemiplegia due to cerebrovascular accident. Turkish. Turkiye Fiziksel Tip ve Rehabilitasyon Dergisi 2010; 56(4):177–181.					x			
(89) Noma T, Matsumoto S, Shimodozono M, Kawahira K. Novel neuromuscular electrical stimulation system for the upper limbs in sub-acute stroke patients: a pilot randomized controlled trial. Brain injury 2014; 28(5–6):725.					x			
(90) Packman-Braun R. Relationship between functional electrical stimulation duty cycle and fatigue in wrist extensor muscles of patients with hemiparesis. Phys Ther 1988 Jan; 68(1):51–56.	x							
(91) Page SJ, Levin L, Hermann V, Dunning K, Levine P. Longer versus shorter daily durations of electrical stimulation during task-specific practice in moderately impaired stroke. Archives of Physical Medicine and Rehabilitation 2012 Feb; 93(2):200–206.					x			
(92) Pandyan AD, Granat MH, Stott DJ. Effects of electrical stimulation on flexion contractures in the hemiplegic wrist. Clin Rehabil 1997 May; 11(2):123–130.	x							
(93) Pandyan AD, Granat MH. Can neuro-muscular electrical stimulation of the wrist extensors facilitate the recovery of arm function in the severely disabled acute stroke patient? Age Ageing 2004; 33 (Suppl 2):ii43.								x
(94) Pandyan AD, Granat MH. Can treatment with upper limb electrical stimulation be justified in the severely disabled acute stroke patient? 9th Annual Conference of the International FES Society 2004 2004.								x
(95) Peurala SH, Pitkanen K, Sivenius J, Tarkka IM. Cutaneous electrical stimulation may enhance sensorimotor recovery in chronic stroke. Clin Rehabil 2002 Nov; 16(7):709–716.	x							
(96) Popovic DB, Popovic MB, Sinkjaer T, Stefanovic A, Schwirtlich L. Therapy of paretic arm in hemiplegic subjects augmented with a neural prosthesis: a cross-over study. Canadian Journal of Physiology and Pharmacology 2004 Aug–Sep; 82(8–9):749–756.					x		?	
(97) Powell J, Pandyan AD, Granat M, Cameron M, Stott DJ. Electrical stimulation of wrist extensors in poststroke hemiplegia. Stroke 1999 Jul; 30(7):1384–1389.				x				
(98) Ring H, Rosenthal N. Controlled study of neuroprosthetic functional electrical stimulation in sub-acute post-stroke rehabilitation. J Rehabil Med 2005 Jan; 37(1):32–36.				x				
(99) Rosewilliam S, Malhotra S, Roffe C, Jones P, Pandyan AD. Can surface neuromuscular electrical stimulation of the wrist and hand combined with routine therapy facilitate recovery of arm function in patients with stroke?. Archives of Physical Medicine and Rehabilitation 2012 Oct; 93(10):1715–21.e1.				x				
(100) Sahin N, Ugurlu H, Albayrak I. The efficacy of electrical stimulation in reducing the post-stroke spasticity: a randomized controlled study. Disability and Rehabilitation 2012; 34(2):151–156.					x			
(101) Sentandreu Mano T, Salom Terradez JR, Tomas JM, Melendez Moral JC, de la Fuente Fernandez T, Company Jose C. [Electrical stimulation in the treatment of the spastic hemiplegic hand after stroke: a randomized study]. Med Clin 2011 Sep 17; 137(7):297–301.					x			
(102) Sheng B, Hui-Chan CW. Treatment of upper-limb paresis by transcutaneous electric nerve stimulation and task-related training during chronic stroke: a randomized placebo-controlled trial. Arch Phys Med Rehabil 2007; 88:E2.								x
(103) Sonde L, Gip C, Fernaeus SE, Nilsson CG, Viitanen M. Stimulation with low frequency (1.7 Hz) transcutaneous electric nerve stimulation (low-tens) increases motor function of the post-stroke paretic arm. Scand J Rehabil Med 1998 Jun; 30(2):95–99.					x			
(104) Sonde L, Kalimo H, Fernaeus SE, Viitanen M. Low TENS treatment on post-stroke paretic arm: a three-year follow-up. Clin Rehabil 2000 Feb; 14(1):14–19.			x					
(105) Sullivan JE, Hurley D, Hedman LD. Afferent stimulation provided by glove electrode during task-specific arm exercise following stroke. Clin Rehabil 2012 Nov; 26(11):1010–1020.				x				
(106) Tarkka IM, Kononen M. Methods to improve constraint-induced movement therapy. NeuroRehabilitation 2009; 25(1):59–68.	x							
(107) Tekeoglu Y, Adak B, Goksoy T. Effect of transcutaneous electrical nerve stimulation (TENS) on Barthel Activities of Daily Living (ADL) index score following stroke. Clin Rehabil 1998 Aug; 12(4):277–280.				x				
(108) Vodovnik L, Kralj A, Stanic U. Recent applications of functional electrical stimulation to stroke patients in Ljubljana. Clin Orthop 1978 1978; NO.131:64–70.	x							
(109) Wang RY, Chan RC, Tsai MW. Functional electrical stimulation on chronic and acute hemiplegic shoulder subluxation. American Journal of Physical Medicine & Rehabilitation 2000; 79(4):385–90; quiz 391–4.	x							
(110) Wang RY, Yang YR, Tsai MW, Wang WT, Chan RC. Effects of functional electric stimulation on upper limb motor function and shoulder range of motion in hemiplegic patients. American Journal of Physical Medicine & Rehabilitation 2002; 81(4):283–290.				x				
(111) Weber DJ, Skidmore ER, Niyonkuru C, Chang CL, Huber LM, Munin MC. Cyclic functional electrical stimulation does not enhance gains in hand grasp function when used as an adjunct to onabotulinumtoxinA and task practice therapy: a single-blind, randomized controlled pilot study. Arch Phys Med Rehabil 2010; 91(5):679–686.					x			
(112) Wong AM, Su TY, Tang FT, Cheng PT, Liaw MY. Clinical trial of electrical acupuncture on hemiplegic stroke patients. American Journal of Physical Medicine & Rehabilitation 1999 Mar–Apr;78(2):117–122.				x				
(113) Wu CW, Seo HJ, Cohen LG. Influence of electric somatosensory stimulation on paretic-hand function in chronic stroke. Archives of Physical Medicine and Rehabilitation 2006 Mar; 87(3):351–357.				x				
(114) Wu FC, Lin YT, Kuo TS, Luh JJ, Lai JS. Clinical effects of combined bilateral arm training with functional electrical stimulation in patients with stroke. IEEE International Conference on Rehabilitation Robotics 2011; 2011:5975367.								x
(115) Yozbatiran N, Donmez B, Kayak N, Bozan O. Electrical stimulation of wrist and fingers for sensory and functional recovery in acute hemiplegia. Clin Rehabil 2006 Jan; 20(1):4–11.					x			

## Appendix 3

### Ongoing studies list

**Table 2:** ᅟ

Title	Trial registration number	Author
Repetitive arm training combined with functional electrical stimulation on upper extremity motor recovery in sub-acute stroke survivors	NCT02267798	Straudi, S.
Treatment of hand dysfunction after stroke	NCT00508521	Daly, J.
Post stroke hand functions: bilateral movements and electrical stimulation treatments	NCT00369668	Cauraugh, JH.
Brain and coordination changes induced by robotics and fes treatment following stroke	NCT00237744	Daly, J.
MyndMove therapy for severe hemiparesis of the upper limb following stroke	NCT02266836	Hebert, DA; Bayley, M.

Table of possibly relevant ongoing trials (searched 31/10/2015)

## Additional files

Additional file 1: Preferred Reporting Items for Systematic Reviews and Meta-Analyses (PRISMA) statement. (DOC 60 kb)
Additional file 2: Table S1. Outcome Measure Definitions Table and References. **Table S2.** Included Study Characteristics Table and References. **Table S3.** Critical Appraisal Table. (DOC 171 kb)
Additional file 3: Figure S1. SMD (95% CI) of Functional Electrical Stimulation (FES) vs control on Activities of Daily Living for sham-controlled trials only. *AMAT* Arm Motor Ability Test; *CAHAI* Chedoke Arm and Hand Activity Inventory; *FIM* Functional Independence Measure; *UEFT* Upper Extremity Function Test; *HFG* Higher Functioning Group; *LFG* Lower Functioning Group. (JPG 85 kb)
Additional file 4: Figure S2. SMD (95% CI) of Functional Electrical Stimulation (FES) vs control on secondary outcomes (functional motor recovery) measured by FMA. **a** FES initiated within two months of stroke **b** FES initiatied after one year of stroke. (JPG 96 kb)
Additional file 5: Figure S3. SMD (95% CI) of Functional Electrical Stimulation (FES) vs control on secondary outcomes (functional motor recovery) for sham-controlled trials only measured by FMA. (JPG 74 kb)

## Abbreviations

ADL: Activities of daily living
BBT: Box and block test
CI: Confidence interval
FES: Functional electrical stimulation
FIM: Functional independence measure
FMA: Fugl-Meyer assessment
IQR: Interquartile range
PRISMA: Prefered Reporting Items for Systematic Review and Meta-Analysis
RCT: Randomised controlled trial
SD: Standard deviation
SMD: Standardised mean difference
