# Supplementary material for: Effectiveness of upper limb functional electrical stimulation after stroke for the improvement of activities of daily living and motor function: a systematic review and meta-analysis
Source: Syst Rev. 2017 Feb 28;6:40. doi: 10.1186/s13643-017-0435-5 (PMC5331643; doi:10.1186/s13643-017-0435-5)
Supplement: Additional file 2: Table S1. — Outcome Measure Definitions Table and References. Table S2. Included Study Characteristics Table and References. Table S3. Critical Appraisal Table. (DOC 171 kb) [file 13643_2017_435_MOESM2_ESM.doc]

**Additional file 2:** Tables

**Table showing outcome measure definitions with references**

| **Outcome Measure** | **Description** |
| --- | --- |
| ***Primary Outcomes*** | ***Activities of Daily Living*** |
| **Motor Activity Log-14: Amount of Use**  **(MAL AOU)^1^** | MAL is an interview technique that assesses subjective reporting of participants on 14 common daily activities involving the upper limb. The Amount of Use (AOU) subscale assigns each patient a score on an 11-point scale according to the amount of use they retain of their more affected arm, as compared to their motor function prior to stroke. |
| **Motor Activity Log-14: Quality of Movement**  **(MAL QOM)^1^** | MAL is an interview technique that assesses subjective reporting of participants on 14 common daily activities involving the upper limb. The Quality of Movement (QOM) subscale assigns each patient a score on an 11-point scale. Their responses are scored according to how well they are now able to use their more affected arm to perform specific functional activities, as compared to their motor function prior to stroke. |
| **Barthel Index Score**  **(BIS)^2^** | Barthel Index is a score based on 16 items which include act ivies of daily living, mobility, cognitive and social functioning. A 4 item subset of the score focuses on items specifically related to activities of daily living that require the upper limb. |
| **Functional independence Measure**  **(FIM)^3^** | FIM is a score, originally derived from the Barthel Index, which considers 18 items related to upper limb requiring activities of daily living. Participant ability to perform each item independently is measured on a 7 point qualitative scale. |
| **Upper Extremity Function Test**  **(UEFT)^4^** | UEFT is a measure of ability to perform common activities of daily living. Participants have to complete as many repetitions of each task as they can in 2 minutes. |
| **Arm Mobility Arm Test**  **(AMAT)^5^** | AMAT assess functional ability to carry out 28 upper limb specific activities involving everyday objects. |
| **Chedoke Arm & Hand Activity Inventory (CAHAI)^6^** | CAHAI is a 7-point quantitative scale that assesses functional recovery of the arm and hand post-stroke across a range of activities of daily living. Activities are scored according to the patient’s ability to complete them, from entirely assisted to totally independent. |
| **Rehabilitation Engineering Laboratory Hand Test: Object Manipulation Subscale (RELHT OS)^7^** | This is a subscale of the full RELHT scale that measures the patient’s ability to pick up and maintain palmar grasp of 9 objects, in a variety of pronated/neutral/supinated postures that mimic activities of daily living. Their ability to perform each task is scored on a points scale then summed to give an overall total. |
| ***Secondary Outcomes*** | ***Functional motor outcomes*** |
| **Fugl-Meyer Assessment**  **(FMA)^8^** | FMA is a 33 item score that assesses movement, reflexes and coordination of the upper limb on a 3 point scale. |
| **Box & Block Test**  **(BBT)^9^** | This test requires participants to grasp and move a small wooden cube over a central barrier in a box and drop it on the other side. The number of boxes moved in 1 minute is then counted. |
| **Motor Assessment Scale: Hand Movement**  **(MAS HM)^10^** | MAS examines 9 areas of motor function and scores them on a 7-point qualitative scale based on participant ability to perform the relevant tasks.  The hand movement (HM) subscale assesses ability to perform various functional movements of the hand, scoring the patient’s overall performance on a scale from 0-6. |
| **Motor Assessment Scale: Upper Arm Function**  **(MAS UAF)^10^** | MAS examines 9 areas of motor function and scores them on a 7-point qualitative scale based on participant ability to perform the relevant tasks.  The Upper Arm Function (UAF) subscale assesses ability to perform various movements of the upper arm at the shoulder joint in supine and standing positions. Performance is overall scored on a scale from 0-6. |
| **Action Research Arm Test**  **(ARAT)^11^** | ARAT qualitatively measures the ability to manipulate objects. It is split into 4 subsections: grasp, grip, pinch and gross movement. |
| **Chedoke McMasters Stroke Assessment**  **(CMSA)^12^** | CMSA score assesses functional ability of the upper limb and each item is scored on a 7 point scale from no voluntary muscle activity to normal movement. |
| **Functional Test for the Hemiplegic Upper Extremity**  **(FTHUE)^13^** | FTHUE is based on 18 activities of daily living involving the upper limb, which are given one of seven functional levels depending on the degree of difficulty performing each activity. |
| **Rehabilitation Engineering Laboratory Hand Test: Block Movement**  **(RELHT BM)^7^** | This is a subscale of the full RELHT scale that measures the patient’s ability to pick up, hold and release 9 wooden blocks of varying surface friction and weight. For each block the patient is scored across a three-point scale according to their ability to perform the task and then a total score calculated. |
| **9-Hole-Peg-Test**  **(9HPT)^14^** | A seated patient is asked to place 9 uniform pegs from a container into holes in a pegboard, then return them to the container, as fast as possible. The time taken to complete the task is recorded. |
| **10-Cup-Moving-Test**  **(10CMT)^15^** | Measures the patient’s ability to close their fingers around a cup, move it a short distance, then release, repeated 10 times. |
| ***Tertiary Outcomes*** | ***Other motor outcomes including muscle tone, force generated and range of active movement*** |
| **Modified Ashworth Score**  **(MAS)^16^** | MAS is a measure of resistance to passive movement (spasticity) of the upper limb, which is rated on a 5 point scale. |
| **Force Generation (Sustained and Maximal)** | This outcome assessed sustained & maximal contraction of individual upper limb muscle groups. Methods varied across studies. |

**Table S1: Outcome Measure Definitions Table & References**. This describes the measurement instruments used in the included studies, for our defined outcome measures. References listed below.

| **Table showing included study characteristics with references**  **Study** | **Methods** | **Participants** | **Intervention** | **Comparator** | **Outcomes** |
| --- | --- | --- | --- | --- | --- |
| **Barker 2009^A1^** | Randomisation: Yes but method not explicit.  Blinding: Single; evaluators were blinded only. | Numbers: 23  - **Intervention**: EMG-triggered stimulation + SMART arm training (n=10)  - **Comparator**: SMART arm training alone (n= 13)  Mean age and gender:  - **Intervention**: 61y, 60% male  - **Comparator:** SMART arm training only: 67y, 85% male  Stroke type: not stated.  Time since stroke: ≥6 months.  Stroke severity: Severe upper limb paresis MRC grade 1-3 for triceps & inability to complete a standardised supported reaching task (push 25g sandbag off table with elbow extended); detectable surface EMG signal from paretic triceps. | Regime: 3 surface electrodes were applied to triceps & forearm. Outcome-initiated EMG-triggered stimulation of triceps.  Threshold voltage initially 50µV, but varied in response to patient’s performance, f= 50Hz, T = 1s ramp up, 5-10s sustained biphasic stimulation (200µs pulse width), 1s ramp down. 10-20s rest between. Stimulation sessions lasted 60 minutes.  This was superadded to 12 concomitant 1-hour SMART arm training sessions in the same 4 week period.  Splint/orthosis: SMART arm: reduces degrees of freedom of movement in wrist & reduces resistance from surface friction.  Home/hospital based: Not stated, delivered by physiotherapists. | Standard care: 12 1h sessions of SMART arm training over 4 week duration, without electrical stimulation.  Splint/orthosis: SMART arm.  Sham tx: None.  Home/hospital based: not stated. | Outcomes:   - Modified Ashworth Score (Upper Arm Function, item 6 subscale) - Motor Assessment Scale (UAF*, HM*, AH* sub-scales)   Lab measures:   - Maximum sustained force generation - Maximum distance reached   Timeframe for measuring outcomes:   - 0 weeks (prior to upper limb training) - 4 weeks (completion of training) - 12 weeks (2 months after training) |
| **Cauraugh 2000^A2^** | Randomisation: Yes but method not explicit.  Blinding: Single; evaluators were blinded only.  Modified crossover design: control subjects to receive electrical stimulation after, but RCT analysis performed mid-study, as represented here. | Numbers: n=11  Mean age and gender: 61 y (SD 9.6y), 45% male.  **Intervention**: n=7  **Comparator**: n=4.  Stroke type: Not stated, 10 right-sided, 1 left-sided hemisphere stroke.  Time since stroke: Minimum >1 year post stroke, mean 3.49y (SD 2.56y) post stroke.  Stroke severity: Chronic upper extremity impairment with a maximum 75% motor recovery (via FMA scoring), but minimum >20 degrees voluntary extension at wrist from 90 degree flexed position. Severity assessed by clinical measures (detailed in outcomes), but data not shown at baseline. | Regime: 2 week duration of outcome-initiated EMG stimulation, plus rehabilitation training sessions for finger and wrist extensors. Surface electrodes were applied to hand/wrist extensors. Before stimulation was applied, stretches were performed on the patients’ hemiparetic arm. A reaction-time and sustained contraction task were both performed after each session.  For electrical stimulation, I= 14-29mA, threshold voltage not stated, f= 50Hz, T = 1s ramp up, 5s sustained biphasic stimulation, 1s ramp down, with 25s rest between successful trials.  Stimulation sessions lasted for 30 successful movement trials, twice per day, 3 times per week for 2 consecutive weeks, totalling 12 sessions of approximately 60 minutes each.  Splint/orthosis: None  Home/hospital based: Not stated. | Standard care:  12 sessions of 30 finger/wrist extensor trials, without stimulation, performed over same duration. Hemiparetic arm was stretched before each session. Reaction-time & sustained contraction tasks were performed after each session.  Splint/orthosis: None.  Home/hospital based: Not stated.  Sham tx: None. | Outcomes: Clinical measures:   - Box and Block Test - Fugl-Meyer Assessment - Motor Assessment Scale   Laboratory measures:   - Reaction time chronometry - Sustained & maximal force generation   Timeframe for measuring outcomes: Both intervention and comparator groups were assessed for clinical & laboratory measures at baseline (i.e. pre-test) and after 12 sessions/ 2 weeks (post-test). |
| **Cauraugh 2002^A3^** | Randomisation: Yes but method not explicit.  Blinding: Not stated. | Numbers: 15  Age and gender: No breakdown of relevant patients given  Stroke type: Not stated, 13 left-sided, 12 right sided hemisphere strokes.  Time since stroke: ≥1 year ago, mean time since stroke 39.1 months.  Stroke severity: Mild-moderate upper extremity hemiparesis, with ≤2 CVAs in same hemisphere. <80% motor recovery as assessed by EMG and forced contraction compared to less affected limb, but >10 degrees of voluntary wrist or finger extension against gravity from a 90 degree flexed posture. | Regime: Unilateral application of EMG-triggered stimulation to the wrist and finger extensors of the more affected limb. This took place for 4 days over a 2 week duration. 30 trials were carried out in 3 sessions on each day, each lasting approximately 90 minutes where patients were asked to extend their wrist/fingers. At the start of each treatment session the more affected limb was stretched.  f= 50Hz, I= 16-29mA, threshold voltage = Initially 50µV, varied in response to patient performance. T= 1s ramp up, 5s sustained biphasic stimulation (pulse width 200µs), 1s ramp down. 25s rest between trials.  Splint/ orthosis: None.  Home/hospital based: Not stated. | Standard care:  Performed same activities as intervention but without electrical stimulation. Attempts to extend fingers/wrist were for 5s, 25s rest between trials, in total for approx. 90 minutes.  Home/hospital based: Not stated.  Sham tx: None. | Outcomes:   - Box and block test - Reaction time chronometry - Sustained & maximal force generation   Timeframe for measuring outcomes: Pre- and post- intervention testing at 0 and 2 weeks. |
| **Cauraugh 2003 (JNS)^A4^** | Randomisation: Yes, computer-generated randomization.  Blinding: Not stated. | Numbers: **Overall:** n=26  **Intervention:** n=20, split into n=10 across two subgroups.  **Comparator:** n=6.  Age and gender: **Overall:** 66.4y (SD 9.7y), 69% male.  **Intervention:**   - 5s stimulation group: 70% male - 10s stimulation group: 70% male   **Comparator:** 67% male  Stroke type: Not stated  Time since stroke: >1 year post stroke, ‘average’ 2.8y (SD 1.9y)  Stroke severity: Not stated, though all patients had partial paralysis on wrists/fingers and difficulty in voluntarily initiating/ controlling extensor movements. | Regime: Electrical stimulation was applied via electrodes at the back of the impaired forearm during 4 sessions over a 2 week period. Both intervention groups performed 3 sets of 30 successful trials of wrist/finger extension, during each 90 minute session, at the same time accompanied by bilateral movement training. The two intervention subgroups were identical in regime, except that one received 5s sustained electrical stimulation; the other 10s sustained electrical stimulation.  Threshold voltage = 50µV initially, but variable in response to patient’s performance, f = 50Hz, I= range 17-28mA, T= 1s ramp up, 5 or 10s sustained biphasic stimulation (Pulse width 200µs), 1s ramp down.  Splint/orthosis: None.  Hospital/home based: Not stated. | Standard care: Controls performed an identical regime over the same 2 week duration, but without electrical stimulation. A rehabilitation trainer supervised as they voluntarily extended wrist/fingers 90 times in each of the 4 total sessions.  Sham tx: None.  Hospital/home based: Not stated. | Outcomes:   - Box and block test - Reaction time chronometry - Sustained & maximal force generation   Timeframe for measuring outcomes: Pre- and post- intervention testing at 0 and 2 weeks. |
| **Cauraugh 2003 (JNNP)^A5^** | Randomisation: Yes- but method not explicit.  Blinding: None | Numbers: n=34 in total. **Intervention**: n=28, receiving neuromuscular stimulation, split into:  - ‘Blocked’ practice schedules of repetitive movements on consecutive trials (n=14)  - ‘Random’ practice schedules of different movements on successive trials (n=14)  **Comparator**: n= 6  Age and gender: **Overall**: mean age 66.1y (48.6-81.2y range); 30 men, 4 women. **Intervention**: Blocked= 12 men, 2 women. Random-group= 13 men, 1 woman  **Comparator**: 5 men, 1 woman  Stroke type: Not stated.  Time since stroke: **Overall** 3.2y.  Stroke severity: Absence of neurological deficits other than hemiparesis, <2 prior strokes. Voluntary wrist/finger extension >10° from a 90° flexed wrist position, <80% motor recovery compared to non-paretic limb. | Regime: Non-EMG-triggered neuromuscular electrical stimulation to the wrist/finger extensors, triceps & anterior/middle deltoid. 3 sets of 30 trials (lasting 90 minutes total) for each of 2 weeks was performed, with 10 successful trials in each set allocated to different movements:   1. Wrist/finger extension 2. Elbow extension 3. Shoulder abduction   The two intervention groups were split according to the blocked or random practice schedule allocated, where the 3 movements were either repetitively performed on consecutive trials or as different movements on successive trials. A minimum of 24h rest was enforced between sessions.  Threshold voltage = 50µV initially, but variable in response to patient’s performance, f = 50Hz, I= range 13-26mA, T= 1s ramp up, 5s sustained biphasic stimulation (Pulse width 200µs), 1s ramp down. 25s rest between trials.  Splint/orthosis: None.  Hospital/home based: Not stated. | Standard care:  Comparator group underwent the same regimen over the same 2 week period, but did not receive stimulation. For 90 mins/day for 4 days in that period, each joint/set of muscles were passively moved and subjects attempted to extend wrist/fingers, extend elbow and abduct the shoulder.  Sham tx: None. Hospital/home based: Not stated. | Outcomes:   - Box and Block Test - Reaction time chronometry - Sustained & maximal force generation   Timeframe for measuring outcomes:  Pre- and post-intervention testing at 0 and 2 weeks. |
| **Chan 2009^A6^** | Randomisation: Yes- but method not explicit.  Blinding: Double, both patients and assessor. | Numbers: **Overall**: 20.  Intervention: n=10  Comparator: n=10  Age and gender: **Intervention**: 46y (±17y), 50% male.  Comparator: 45 y (±16y), 60% male.  Stroke type: Not stated.  Time since stroke: ≥6 months.  Stroke severity: First episode of stroke, score of 0 in finger mass extension subitem of the Fugl-Meyer Assessment, GCS 15. | Regime: 15 sets of 90 mins daily, consisting of:   - 10 mins stretching activities - 20 mins electrical stimulation and 4 functional tasks - 60 mins conventional therapy training.   Voltage threshold not specified, I= not stated, f= 40Hz, T= 3s ramp up, 3s sustained stimulation (200µs pulse width), 2s ramp down.  Splint/orthosis: Wrist extension splint, keeping wrist extended at 15°.  Hospital/home based:  Not stated. | Standard care:  Same regime as intervention, but sub-threshold electrical stimulation given instead.  Sham tx: Yes, placebo trial, sufficient to provide sensory stimulation to comparators, but below the threshold for motor stimulation.  Hospital/home base: Not stated. | Outcomes: Primary:   - Functional Test for the Hemiplegic Upper Extremity (FTHUE) - Fugl Meyer Assessment - Measurement of forward reaching distance - Sustained & maximal force generation - Grip power - Active range of active motion (ROAM) of wrist extension in affected hand.   Secondary:   - Functional Independence Measure (FIM) - Modified Ashworth Score (MAS)   Timeframe for measuring outcomes: Upper limb function tests performed before/after 15 sessions of training. |
| **Duarte 2011^A7^** | Randomisation: Yes- via ‘secret assignment’ with black and white balls.  Blinding: Double-blinding of both patients and medics evaluating them | Numbers: n=24 **Intervention** n=13, **comparator** n=11.  Age and gender: **Intervention**: 8 men, 5 women; mean age 57.7 (SD 13.4).  **Comparator**: 8 men, 3 women; mean age 55.2 (SD 16.3).  **Overall**: 16 men, 8 women; mean age 56.6 (SD 14.5)  Stroke type: **Intervention**: 11 ischaemic, 2 haemorrhagic.  **Comparator**: 7 ischaemic, 4 haemorrhagic.  **Overall**: ischaemic 18, haemorrhagic 6.  Time since stroke: **Intervention**: 3.5 years (3.64 SD)  **Comparator**: 2.7 years (1.65 SD)  **Overall**: 3.1 years (2.88 SD)  Stroke severity: Brunnstrom classification scores 3-4 for upper limb motor dysfunction. Spasticity of wrist/hand flexion equivalent to Modified Ashworth Scale score ≥ 3. | Regime: Intervention group received 12 sessions of electrical stimulation, 30mins duration each, electrodes applied to distal extensor muscles in the affected upper limb.  Threshold voltage not stated, f= 50Hz, I=50-90mA, T= 2s ramp up, 6s biphasic stimulation (pulse width 300 µs), 2s ramp down.  Splint/orthosis: None.  Hospital/home based: Hospital-based, outpatients setting. | Standard care: Yes- 200 units botulinum toxin type A, injected into flexor muscles of wrist/fingers.  Comparators also received 12 sessions placebo FES (sham) by way of placing electrodes away from motor points. This resulted in cutaneous stimulation above sensory threshold, but below motor threshold.  Sham tx: Yes.  Hospital/home based: Not stated. | Outcomes:  Motor function:   - Fugl-Meyer Assessment - Medical Research Council Scale (measure of muscle strength) - Motricity Index for Motor Impairment after stroke (Arm test) - Nicholas Manual Muscle Tester (NMMT)   Functional capacity of upper limb   - Chedoke Arm and Hand Activity Inventory (CAHAI) - Box and Block Test (BBT)   Spasticity measures:   - Modified Ashworth Scale (MAS)   Timeframe for measuring outcomes: 4 and 16 weeks post injection of botulinum toxin. |
| **Francisco 1998^A8^** | Randomisation: Yes; computer-generated random number table.  Blinding: Single blinding of assessor. | Numbers: 9 patients in total, **Intervention**: n=4  **Comparator**: n=5  Age and gender:  **Intervention:** 60.3y (SD 15.6y), 50% male  **Comparator:** 69.6y (SD 16.2y), 40% male  Stroke type: Non-haemorrhagic.  Time since stroke: <6 weeks  Stroke severity:  MRC power score in paretic wrist extensors <3/5; no other neurologic co-morbidities that would have impaired strength in the affected upper extremity, no sensory loss in the forearm. | Regime: 2 sets of 30 minute rehabilitation sessions daily, 5 times per week with EMG-triggered neuromuscular stimulation for the duration of the patient’s rehabilitation stay (exact duration is not stated.)  Voltage threshold= 5µV initially, but gradually increased as each patient’s number of sessions progressed, f= 20-100Hz, I= 0-60mA, T= Biphasic sustained stimulation for 5s on, (200µs square pulse width), 5s off.  Splint/orthosis: None.  Hospital/home based: Hospital based. | Standard care:  Comparators received everything interventions did, except EMG- triggered stimulation. Stroke rehab for both groups included neuromuscular re-education, ROM exercises, strengthening etc.  Sham tx: None.  Hospital/home based: Hospital-based care. | Outcomes:   - Fugl-Meyer Assessment: upper extremity items - Functional Independence Measure (FIM): Items relating to feeding/ grooming/ upper body dressing   Timeframe for measuring outcome: At study entry and discharge. |
| **Hara 2008^A9^** | Randomisation: Yes, by computer-generated randomisation.  Blinding: None. | Numbers: **Overall** n=20. Split into **intervention** groups A & B (n=5, n=5) according to anatomical placement of electrodes. These are matched against two **comparator** groups (n=5, n=5).  Age and gender: **Intervention**: mean age 56, (range 24-77) 80%  **Comparator**: Mean age 60.5, 6 men, 4 women  Stroke type: Not stated.  Time since stroke:  **Intervention** group: 13 months (range 12-16 months)  **Comparator**: 13 months.  Stroke severity: Absence of neurological deficit other than hemiplegia, ≤2 strokes on same side of brain, upper extremity Stroke Impairment Assessment Set (SIAS) score ranging from 0-5. Passive range of motion on affected side of 0-45° wrist extension, 0-140° shoulder joint flexion. Some voluntary wrist/ finger/ shoulder movement. | Regime: 30-60 minute sessions of EMG-triggered electrical stimulation, 5 days per week, carried out by the patient, over a 5 month period. This was gradually increased over the first 10 days to a maximum duration of 1h sessions, with regular follow-up visits to ensure adherence to treatment protocol. Additionally patients were supervised once per week by a rehabilitation trainer.  The intervention group was split according to the anatomical placement of electrodes for stimulation:   - Group A: Electrodes placed on posterior forearm - Group B: Electrodes placed on anterior deltoid/ medial aspect upper arm   Voltage threshold= not specified, f= not stated, I= not stated, T= Duration of biphasic rectangular stimulation (50µs pulse width) not stated.  Splint/orthosis: None.  Hospital/home based: Home-based. | Standard care: Comparators followed the same procedure as intervention for the outpatient rehabilitation program, but no stimulation given. Care not explicitly stated. Occupational therapists apparently worked with all patients towards ‘individualized goals.’  Sham tx: None.  Hospital/home based: Home-based. | Outcomes:   - Active Range of Motion (ROM) - Modified Ashworth Scale (MAS) - 10-Cup-Moving-Test (10CMT) - 9-Hole-Peg Test (9HPT) - EMG root mean square values (ERMS)   Timeframe for measuring outcomes:  5 months after starting therapy, when the therapy ended. |
| **Mangold 2009^A10^** | Randomisation: Yes, computer-generated randomization list.  Blinding: Not stated. | Numbers: **Overall:** n=23  **Intervention:** n=12  **Comparator:** n=11  Age & gender:  **Intervention:** 57.5y (SD=16.7y), 83% male.  **Comparator:** 62 y (SD= 16.2y), 64% male.  Stroke type: Infarct or haemorrhage, localised from cortex to brainstem.  Time since stroke: 2- 18 weeks.  Stroke severity:  Severe hemiparesis/ complete hemiplegia of affected arm or hand, with maximum value 3 on the Chedoke McMaster Stroke Assessment (CMSA) for arm and hand. Able to sit in a wheelchair/chair with no contact to the back rest. | Regime: 4  week study. 3-5 occupational therapy sessions (45 mins) were performed each week, involving repetitive grasping functions. In the intervention group, 12 of these sessions across the 4 week period also involved application of electrical stimulation. Electrodes were placed to aid movements as follows:   - Proximally: anterior deltoid, triceps - Distally: finger flexors/extensors   Voltage threshold not specified, I= not stated, f= 25Hz, T= Duration of stimulation not specified, but pulse width on application varied 0 - 250µs.  Splint/orthosis: None.  Home/hospital based: Not stated. | Standard care: Occupational therapy.  Sham tx: None.  Home/hospital based: Not stated. | Outcomes:  Primary:   - Extended Barthel Index subscore for Activities of Daily Living (ADL) - Chedoke McMaster Stroke Assessment (CMSA) of hand/arm function/shoulder pain   Secondary:   - Modified Ashworth Scale (MAS): resistance to passive movement in affected joint   Timeframe for measuring outcomes: Baseline measures 8 days & 1 day before start of 4 week program. Post-treatment assessment performed after 4 week program. |
| **McCabe 2015^A11^** | Randomisation: Yes - but method not explicit.  Blinding: Single- assessor blinded to group assignment of each subject. | Numbers: **Overall:** n=23  **Intervention**: n=12  **Comparator**: n=11  Age and gender: **Intervention**: 7 male, 5 female; age ranges: 3 aged 21-49, 9 aged 50-81.  **Comparator**: 6 male, 5 female; age ranges: 2 aged 21-49, 9 aged 50-81.  Stroke type: Not stated.  Time since stroke: All >1y.  **Intervention**: 10 patients 1-3y post-stroke, 2 patients ≥4 years post-stroke.  **Comparator**: 8 patients 1-3y post-stroke, 3 patients ≥4 years post-stroke.  Stroke severity: Persistent (>1 year) upper extremity impairment, single unilateral stroke, sufficient function to be able to perform ADLs & at least trace muscle contraction in the wrist extensors. | Regime: 3.5h per day of motor learning, plus 1.5h per day functional electrical stimulation training, for 5 days per week, up to a total of 60 sessions.  Splint/orthosis: None.  Hospital/home based: Hospital-based (i.e. states ‘Medical centre.’) | Standard care: 5hrs per day of motor learning, 5 days per week up to a total of 60 sessions, but no functional electrical stimulation.  Sham tx: None  Hospital/home based: Not stated. | Outcomes:  Primary:   - Arm Motor Ability Test (AMAT)   Secondary:   - Fugl Meyer Assessment (FMA): upper limb. - (2 subscales of AMAT also available)   Timeframe for measuring outcomes: Not explicit but states all patients received 5 sessions/week up to a total of 60 sessions, inferring measurement of outcomes after 12 weeks total therapy- but no indication whether or not this is continuous. |
| **Popovic 2002^A12^** | Randomisation: Yes- but method not explicit.  Blinding: Not stated. | Numbers: **Overall:** n= 16  **Intervention:** n=8  **Comparator:** n=8  Age and gender: 57.8y (mean), gender not stated.  Stroke type: Not stated.  Time since stroke: 3-11 weeks.  Stroke severity: Unclear. | Regime: 3 weeks electrical stimulation for 30 minute daily sessions whilst performing functional activities involving reach and use. The intervention group was split according to high and low functioning subgroups according to the patients’ degree of disability at the start of the trial, but their treatment was identical.  Voltage threshold not specified, f=50 Hz, I=15-45mA, T=300µs pulse width, but exact length of sustained stimulation not specified.  Splint/orthosis: No  Home/hospital based: Not stated. | Standard care: Same as intervention, except no electrical stimulation. Comparators were similarly split into high & low functioning subgroups.  Hospital/home based: Not stated.  Sham tx: None. | Outcomes:   - Upper Extremity Function Test (UEFT)   Timeframe for measuring outcomes: At baseline & every 2 months thereafter for 18 months. |
| **Popovic 2003^A13^** | Randomisation: Yes, by random generator.  Blinding: Evaluators blinded. | Numbers: **Overall:** n= 28  **Intervention:** n= 14  **Comparator:** n=14  Age and gender: **Overall:** Mean 59.9y (±9.3y), gender not stated.  Stroke type: 23 ischaemic, 5 haemorrhagic  Time since stroke: Ranged from 4-11 weeks.  Stroke severity: Unclear. | Regime: 3 weeks electrical stimulation for 30 minute daily sessions, in addition to conventional therapy.  Voltage threshold not specified, f= 50 Hz, I= 20-45mA, T= 200 µs pulse width, duration intended to mimic apprehension, grasp and release of a normally functioning hand.  Splint/orthosis: Neural prosthesis.  Home/hospital based: Hospital. | Standard care: Conventional daily therapy, without electrical stimulation.  Hospital/home based: Hospital- based.  Sham tx: None. | Outcomes:  - Upper Extremity Functioning Test (UEFT)  - Drawing Test (DT)  - Modified Ashworth Scale (MAS) of key paretic arm muscles  - Reduced Upper Extremity Motor Activity Log (RUE/MAL)  Timeframe for measuring outcomes: At baseline & at the end of 3 weeks stimulation, 6, 13 and 26 weeks. |
| **Shimodozono 2014^A14^** | Randomisation: Yes, computer-generated blocked randomisation sequence.  Blinding: Assessors blinded, participants blinded to hypotheses of the study. | Numbers: **Overall:** n=18  **Intervention:** n=9  **Comparator:** n=9  Age and gender:  **Intervention:** 61.1y (SD±11.3y), 67% male.  **Comparator:** 61.9y (SD±13.6y), exercise+ stimulation 61.1±11.3y. 100% male.  Stroke type: **Intervention:** 7 infarction, 2 haemorrhagic.  **Comparator:** 5 infarction, 4 haemorrhagic.  Time since stroke: 3-13 weeks.  Stroke severity: Severe arm impairment with Fugl-Meyer Assessment (FMA) score ≤20. | Regime: 4 weeks of 40 mins exercise & electrical stimulation, for 5 days a week, involving both active and passive (therapist-assisted) movement of:   - Shoulder - Elbow - Wrist   Additionally, 30 mins of dexterity-related training after each treatment session was performed daily. Standard inpatient rehabilitation was also adhered to.  Voltage threshold for stimulation is not specified, f=20Hz, I= not stated, T= Applied continuously during exercise, with symmetrical biphasic waveform (250µs pulse width.)  Splint/orthosis: No.  Home/hospital based: Hospital. | Standard care: inpatient rehabilitation programme  Sham tx: None.  Hospital/home based: Hospital. | Outcomes: Primary:   - Fugl-Meyer Assessment   Secondary:   - Range of active movement (ROAM) - Modified Ashworth Scale (MAS)   Timeframe for measuring outcomes: Start of trial and at 4 weeks. |
| **Shin 2008^A15^** | Randomisation: Yes- but method not explicit.  Blinding: Not stated. | Numbers: **Overall:** n=14  **Intervention:** n=7  **Comparator:** n=7  Age and gender: **Overall:** 57.6y (SD±9.5y), 12 male (85.7%)  Stroke type: 8 ischaemic, 6 haemorrhagic.  Time since stroke: **Overall:** range 13-35 months.  **Intervention:** Mean 18.6 months  **Comparator:** Mean 19.7 months  Stroke severity: < grade 2 Modified Ashworth Scale score & able to voluntarily extend >20° against gravity from 90° flexed posture at MCP joint of 3^rd^ finger. | Regime: 2 x 30 minute sessions of EMG-triggered electrical stimulation per day, 5 times a week for 10 weeks.  Voltage threshold not specified, f=35Hz, I=10-20mA, T= 0.1s ramp up, 5s symmetrical rectangular biphasic constant stimulation (pulse width 200 µs), 2s ramp down. 4s rest between trials.  Splint/orthosis: No.  Home/hospital based: Not stated. | Standard care: Same regime as intervention, without electrical stimulation.  Sham tx: None.  Hospital/home based: Not stated. | Outcomes:   - Box & Block Test (BBT) - Strength - Accuracy index - Maximum force generated   Timeframe for measuring outcomes: At 0 and 10 weeks of treatment. |
| **Shindo 2011^A16^** | Randomisation: Yes, using computer-generated list.  Blinding: Assessors blinded. | Numbers: n=20, **Intervention:** n=10, **Comparator:** n=10  Age and gender:  **Intervention**: 58.2y (SD±18.6y)  **Comparator:** 57.9y (SD±9.7y). 15 male (62.5%)  Stroke type: 14 ischaemic, 6 haemorrhagic  Time since stroke: <60 days.  Stroke severity: 1^st^ episode unilateral supratentorial stroke, unable to fully extend paretic fingers. Has a passive range of wrist extension >0° & >-10° for MCP joint extension, MMSE >23. | Regime: 3 week duration of integrated volitional electrical stimulation (IVES) with a splint, applied for 8h daily. This is a form of EMG-triggered electrical stimulation. Alongside this, a standard rehabilitation program was followed, involving 2h additional training per day, 5 days per week.  The specific regime of stimulation used however is not stated.  Splint/orthosis: Yes- wrist splint.  Home/hospital based: Not stated. | Standard care: Same as intervention; all wore wrist splint & underwent same rehabilitation program, but no electrical stimulation.  Sham tx: No.  Hospital/home based: Not stated. | Outcomes:   - Fugl-Meyer Assessment (FMA) - Action Research Arm Test (ARAT) - Modified Ashworth Scale (MAS) - Motor Activity Log 14 (MAL)   Timeframe for measuring outcomes: At pre and post treatment. |
| **Tarkka 2011^A17^** | Randomisation: Yes- but method not explicit.  Blinding: Not stated. | Numbers: n=20  **Intervention:** n=10  **Comparator:** n=10  Age and gender: **Overall:** 53±6, 13 (65%) males  Stroke type: 10 ischaemic, 10 haemorrhagic.  Time since stroke: All ≥6 months, mean 2.4y (SD±2y)  Stroke severity: Severe functional deficit in affected upper limb, no major cognitive difficulties. | Regime: 2 x 30 minute treatment sessions of functional electrical stimulation per day, 5 days per week for a total of 2 weeks duration. Each session also incorporated hand/ arm rehabilitation exercises & functional tasks. Electrodes were applied to wrist/hand flexors & extensors.  Specific regime of electrical stimulation used is not stated.  Splint/orthosis: No.  Home/hospital based: Not stated. | Standard care: Rehabilitation regime as per intervention, except no stimulation.  Hospital/home based: Not stated.  Sham tx: No. | Outcomes:   - Wolf Motor Function test (WMFT): assesses hand and arm voluntary motor behaviour - Transcranial magnetic brain stimulation system for assessment of corticospinal excitability   Timeframe for measuring outcomes: At baseline before treatment, immediately after the 2 week regime and at 6 months follow up. |
| **Thorsen 2013^A18^** | Randomisation: Yes- but method not explicit.  Blinding: Double: assessor and subjects blinded. | Numbers: **Overall:** n=8  **Intervention:** n=4  **Comparator:** n=4  Age and gender: **Intervention:** Median age 39y (IQR 37,50y)  **Comparator:** Median age 57y (IQR 49,62y)  Stroke type: Not specified.  Time since stroke: All >2 months minimum. **Intervention**: Median 8 months (IQR 5, 9), **Comparator:** Median 9 months (IQR 6, 38).  Stroke severity: Paresis of the upper limb with compromised functionality but some residual proximal control. | Regime: Total of 25 x 45 min sessions, ranging from 3-5 sessions per week.  Each session involved application of EMG-triggered electrical stimulation, in addition to standard physiotherapy.  Voltage threshold not specified, f= not stated, I=10-20mA, T= Biphasic 300µs rectangular impulses, 300µs inter-phase interval, 16.6 pulses per second fixed repetition rate.  Splint/orthosis: No.  Home/hospital based: Not stated. | Standard care: Standard physiotherapy, plus sham (i.e. subthreshold) electrical stimulation.  Home/hospital based: Not stated.  Sham tx: Yes. | Outcomes: Primary:  - ARAT  Secondary:  - Individually Prioritized Problem Assessment (IPPA)  Timeframe for measuring outcomes: Before and immediately after treatment, plus follow up of ARAT score at 3 months. |
| **Thrasher 2008^A19^** | Randomisation: Yes using sealed envelopes.  Blinding: Assessor blinded to intervention.  Second part of study used a convenience sample to assess a chronic hemiplegia stroke group. | Numbers: **Overall:** n=21  **Intervention:** n=10  **Comparator:** n=11  Age and gender:  **Intervention:** 57y (SD±14.7y)  **Comparator:** 58y (SD±19.7y)  Stroke type: Not stated.  Time since stroke: 2-7 weeks.  Stroke severity: Chedoke-McMaster Stages of Motor Recovery (CMSMR) score of 1-2 for combined hand and arm function. | Regime: 5 sessions per week electrical stimulation plus conventional therapy for 12-16 weeks, with each session lasting 45 minutes. Conventional therapy included occupational and physiotherapy.  Voltage threshold not specified, f=40Hz, I= 10-50mA.  Splint/orthosis: No.  Home/hospital based: Not stated. | Standard care: Conventional therapy as per intervention, except no stimulation.  Home/hospital based: Not stated.  Sham tx: No. | Outcomes:   - Rehabilitation Engineering Laboratory Hand Function Test (RELHFT) - Functional Independence Measure (FIM)* - Barthel Index (BI) - Chedoke-McMaster Stages of Motor Recovery (CMSMR) - Fugl-Meyer Assessment (FMA)*   Timeframe for measuring outcomes: Immediately before and after treatment. |
| **Yun 2011^A20^** | Randomisation: Yes using random number table.  Blinding: Not mentioned. | Numbers: **Overall:** n=40, **Intervention:** n=20, **Comparator:** n=20  Age and gender: **Intervention:** Mirror therapy+ stimulation 65.9y (SD±10.8y), 60% male.  **Comparator:** Mirror therapy only 63.1y (SD±7.3y), 70% male.  Stroke type: 31 ischaemic, 9 haemorrhagic.  Time since stroke: **Intervention:** 25.6±14.4, **Comparator:** 23.9±10.5.  Stroke severity: Without cognitive impairment and no neurological deficit other than hemiparesis. | Regime: 30 minute sessions for 5 days a week, over a total of 3 weeks. Each session incorporated mirror therapy and electrical stimulation application. Electrodes applied to wrist/finger extensors.  No voltage threshold specified, f=35Hz, I=30-70mA, T=Stimulation was applied for 5s (250µs amplitude), 5s rest between trials.  Splint/orthosis: No.  Home/hospital based: Not stated. | Standard care: Same regime as intervention, except no stimulation.  Home/hospital based: Not stated.  Sham tx: No. | Outcomes:   - Fugl-Meyer Assessment (FMA) - Modified Ashworth Score (MAS)   Timeframe for measuring outcomes: Before & after treatment program. |

Table S2: Included Study Characteristics Table & References. *denotes that data was derived from original data set. References listed below.

| **Study** | Random sequence generation | Allocation concealment | Blinding of participants and personnel | Blinding of outcome assessment | Incomplete outcome data | Selective reporting | Other sources of bias |
| --- | --- | --- | --- | --- | --- | --- | --- |
| Barker 2009^A1^ | Unclear | Unclear | High | Low | Low | Unclear | Low |
| Cauraugh 2000**^A2^** | Unclear | Unclear | High | Unclear | Unclear | Unclear | Low |
| Cauraugh 2002^A3^ | Unclear | Unclear | High | Unclear | Unclear | Unclear | Low |
| Cauraugh 2003 (JNS)^A4^ | Low | Low | High | Unclear | Unclear | Unclear | Low |
| Cauraugh 2003 (JNNP)^A5^ | Unclear | Unclear | High | Unclear | Low | Unclear | Unclear |
| Chan 2009^A6^ | Unclear | Unclear | Low | Low | Low | Unclear | Low |
| Duarte 2011^A7^ | Low | Unclear | Low | Low | Low | Unclear | Low |
| Francisco 1998^A8^ | Low | Unclear | High | Unclear | High | Unclear | Low |
| Hara 2008^A9^ | Low | Unclear | High | Low | Low | Unclear | Low |
| Mangold 2009^A10^ | Low | Unclear | High | Unclear | Low | Unclear | Low |
| McCabe 2015^A11^ | Unclear | Unclear | High | Unclear | Low | Unclear | Low |
| Popovic 2002^A12^ | Unclear | Unclear | High | Unclear | Low | Unclear | Low |
| Popovic 2003^A13^ | Low | Unclear | High | Low | Low | Unclear | Low |
| Shimodozono 2014^A14^ | Low | Unclear | High | Low | Low | Unclear | Low |
| Shin 2008^A15^ | Unclear | Unclear | High | Unclear | Unclear | Unclear | Low |
| Shindo 2011^A16^ | Low | Unclear | High | Low | Low | Unclear | Low |
| Tarkka 2011^A17^ | Low | Low | High | Unclear | Unclear | Unclear | Low |
| Thorsen 2013^A18^ | Low | Unclear | Low | Low | High | Unclear | Low |
| Thrasher 2008^A19^ | Low | Low | High | Low | Unclear | Unclear | Low |
| Yun 2011^A20^ | Low | Unclear | High | Unclear | Unclear | Unclear | Low |

**Table showing critical appraisal of included studies**

**Table S3: Critical Appraisal Table**
