# Supplementary figures and images for: Effectiveness of upper limb functional electrical stimulation after stroke for the improvement of activities of daily living and motor function: a systematic review and meta-analysis
Source: Syst Rev. 2017 Feb 28;6:40. doi: 10.1186/s13643-017-0435-5 (PMC5331643; doi:10.1186/s13643-017-0435-5)

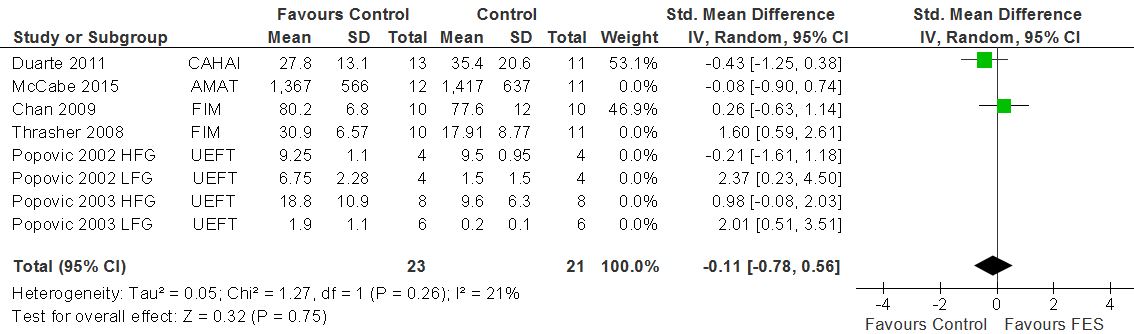

Supplement: Additional file 3: Figure S1. — SMD (95% CI) of Functional Electrical Stimulation (FES) vs control on Activities of Daily Living for sham-controlled trials only. AMAT Arm Motor Ability Test; CAHAI Chedoke Arm and Hand Activity Inventory; FIM Functional Independence Measure; UEFT Upper Extremity Function Test; HFG Higher Functioning Group; LFG Lower Functioning Group. (JPG 85 kb) [file 13643_2017_435_MOESM3_ESM.jpg]

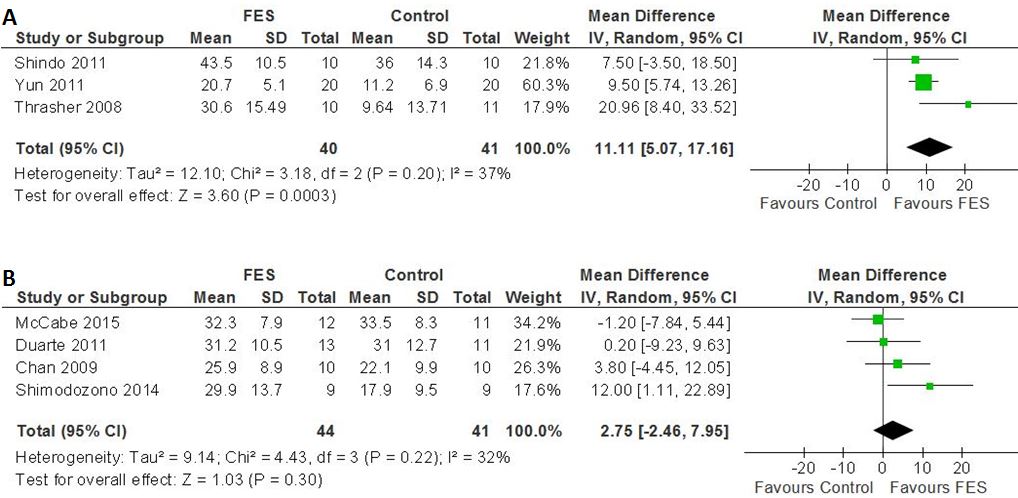

Supplement: Additional file 4: Figure S2. — SMD (95% CI) of Functional Electrical Stimulation (FES) vs control on secondary outcomes (functional motor recovery) measured by FMA. a FES initiated within two months of stroke b FES initiatied after one year of stroke. (JPG 96 kb) [file 13643_2017_435_MOESM4_ESM.jpg]

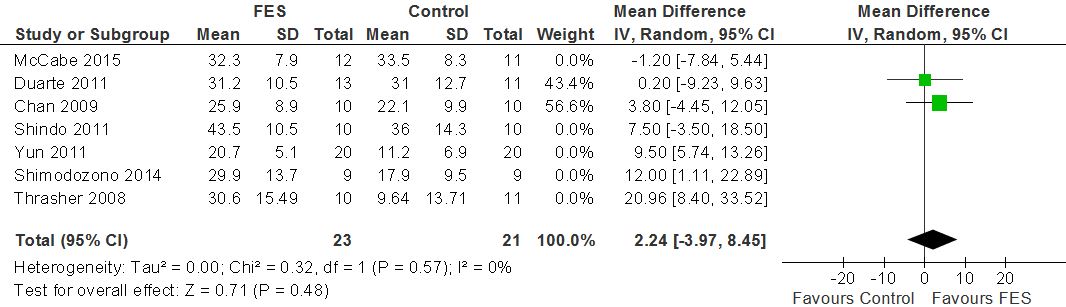

Supplement: Additional file 5: Figure S3. — SMD (95% CI) of Functional Electrical Stimulation (FES) vs control on secondary outcomes (functional motor recovery) for sham-controlled trials only measured by FMA. (JPG 74 kb) [file 13643_2017_435_MOESM5_ESM.jpg]
